# CDC123 is an ATPase that modulates mRNA translation and the integrated stress response by regulating eIF2 complex assembly

**DOI:** 10.1016/j.jbc.2025.111116

**Published:** 2025-12-27

**Authors:** Anthony L. Erb, Sara K. Young-Baird

**Affiliations:** 1 Department of Biochemistry and Molecular Biology, Uniformed Services University, Bethesda, Maryland, USA; 2 Henry M. Jackson Foundation for the Advancement of Military Medicine, Inc., Bethesda, Maryland, USA

**Keywords:** translation control, protein synthesis, integrated stress response, translation initiation, cellular stress

## Abstract

Hyperactivation and hypoactivation of the integrated stress response (ISR) results in impaired regulation of global and mRNA-specific translation in multiple disease contexts. During the ISR, specific stress-sensing kinases modulate translation by regulating the activity of the heterotrimeric eukaryotic translation initiation factor eIF2. Here, we identify the chaperone CDC123, which promotes eIF2 biogenesis, as a novel regulator of the ISR. We find that impaired CDC123 activity reduces eIF2 complex assembly, promoting the translational and cellular outcomes of the ISR through a noncanonical mechanism. Pharmacological or genetic strategies are sufficient to rescue the translational defects associated with impaired CDC123 activity. In addition, we report functional insights into eIF2 heterotrimer formation and provide the first evidence that CDC123-mediated eIF2 complex assembly may be regulated by ATP hydrolysis. These data emphasize the essential contribution of eIF2 biogenesis in mRNA translation regulation, and highlight CDC123 as a possible therapeutic target in the treatment of ISR-related diseases.

Protein synthesis is dynamic and is regulated in response to a wide variety of intracellular and extracellular cues. For example, cells modulate protein synthesis during stress, adapting gene expression programs and metabolism to address cell stress-inducing stimuli. Activity of the eukaryotic translation initiation factor eIF2 serves as a central hub to regulate protein synthesis during cellular stress, mediating the translational outcomes of the integrated stress response (ISR) (1, 2). eIF2 is a heterotrimeric complex, composed of eIF2α, β, and γ subunits. eIF2 associates with GTP and initiator methionyl tRNA (Met-tRNA_i_^Met^) to form the active eIF2, or eIF2 ternary complex (TC), that facilitates translation start site selection along with the 40S ribosomal subunit (3). GTP hydrolysis by eIF2 after start site selection releases the inactive eIF2-GDP binary complex from the ribosome, which must be recycled to eIF2-GTP for another round of protein synthesis to occur. Importantly, during cellular stress, specific stress-sensing kinases phosphorylate the α subunit of eIF2 on Ser51 (eIF2α-P) (1). Phosphorylated eIF2 inhibits the activity of the eIF2 guanine nucleotide exchange factor (GEF), eIF2B, resulting in a decrease in GDP for GTP exchange and ultimately reducing active eIF2 levels to trigger the ISR. Importantly, eIF2 is thought to be ∼10-fold more abundant than eIF2B, rendering eIF2B sensitive to even small changes in the cellular levels of eIF2α-P (4, 5). A reduction in eIF2 TC through this mechanism results in two downstream translational consequences: (1) a decrease in global or general mRNA translation and (2) a selective increase in the synthesis of stress-responsive factors (2). These include the transcription factors ATF4 and CHOP, and the feedback regulator GADD34 that targets PP1c to dephosphorylate eIF2α-P, restoring protein synthesis after stress alleviation (6, 7, 8, 9, 10).

Although the ISR is thought of as a stress-adaptive pathway in most biological contexts, both hyperactivation and hypoactivation of the ISR have been implicated in human disease (11). For example, mutations in the eIF2γ subunit reduce active eIF2 levels and cause the neurodevelopmental disease MEHMO syndrome (12, 13). Reduced active eIF2 levels in this setting cause hyperactivation of the ISR, including chronically low levels of global protein synthesis and hyperexpression of stress-responsive proteins that ultimately result in impaired cell growth and differentiation of MEHMO patient derived cells (14). Conversely, loss-of-function mutations in the endoplasmic reticulum (ER) stress-sensing eIF2 kinase, PERK, reduce eIF2α-P and activation of the ISR, causing Wolcott-Rallison syndrome (WRS) that is characterized by early onset diabetes and multiple epiphyseal dysplasia (15, 16). Thus, the regulation of active eIF2 levels and fine-tuning of ISR signaling are essential for cell function and human physiology.

Although the modulation of eIF2 activity through eIF2α-P has been a dominant focus of the field (1, 2, 11, 17, 18), regulated formation of the eIF2 heterotrimer also ultimately dictates the level of active eIF2 available in the cell. The protein chaperone CDC123 plays a central role in eIF2 biogenesis and is essential for protein synthesis, cell proliferation, and cell viability (19, 20, 21, 22). CDC123 promotes assembly of the eIF2α and eIF2γ subunits, while eIF2β binds to eIF2γ in a CDC123-independent manner (22, 23, 24, 25). Comparative sequence analysis of CDC123 with corroborating structural and biochemical studies have defined CDC123 as an ATP-binding protein with a divergent ATP-grasp fold (23, 26, 27). The atypical nature of the ATP-binding fold and divergence from other ATP-grasp proteins in the catalytic module of CDC123 has limited our understanding of CDC123 enzymatic activity (26). For instance, whether CDC123 can function as an ATPase and how CDC123 ATP binding/hydrolysis impacts eIF2 biogenesis and ISR activation remains unknown.

In this report, we define CDC123 as an ATPase and address the mechanism of CDC123-regulated eIF2 biogenesis. We propose that dysregulated CDC123 ATP hydrolysis may reduce eIF2 complex assembly and show that a mutation in the CDC123 ATP-binding pocket impairs eIF2α and eIF2γ association, promoting the translational and cellular outcomes of the ISR. Reduced CDC123 activity is sufficient to maximally induce the synthesis of the ISR factor, ATF4, through an upstream open reading frame (uORF)-mediated mechanism that is independent of increased eIF2α-P. Finally, we show that pharmacological or genetic strategies to increase the levels of active eIF2 effectively rescue the translational defects associated with impaired CDC123 activity. Our work thus reveals the essential contribution of CDC123-mediated eIF2 biogenesis in mRNA translation regulation and suggests that modulation of CDC123 ATPase activity may be a possible future therapeutic avenue in the treatment of ISR-related diseases.

## Results

### CDC123-A109 is a highly conserved residue located in the CDC123 ATP-binding pocket and is critical for cell growth

**Figure 1:**
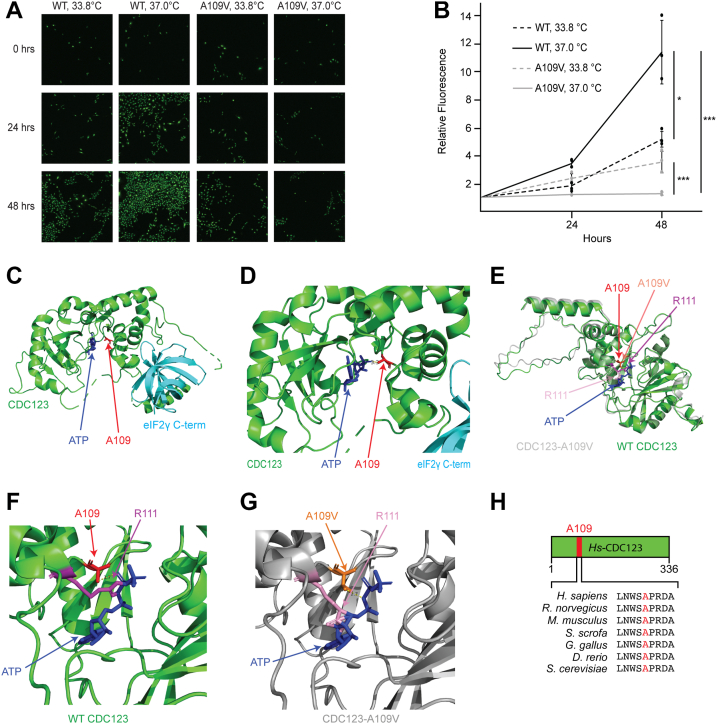
**CDC123-A109 is an evolutionarily conserved residue located in the CDC123 ATP-binding pocket and is important for cell proliferation**. *A*, representative fluorescence images of WT and CDC123-A109V cells grown at 33.8 °C or 37 °C for 0, 24, or 48 h. Cells were stained with CyQUANT Direct Detection Reagent (*green*). *B*, quantification of relative fluorescence from CyQUANT Direct Detection Reagent stained WT and CDC123-A109V cells grown at 33.8 °C or 37 °C for up to 48 h. Average fluorescence with standard deviation (SD) error bars are presented relative to the time 0 fluorescence measurement for each cell line and temperature. Statistical comparisons between groups were conducted using a one-way ANOVA followed by a post hoc Tukey’s test. Significance is indicated by ∗*p* < 0.05, ∗∗∗*p* < 0.0005 (n = 3 biological replicates). *C*, *ribbon* representation of the human eIF2γ C terminus in complex with human CDC123•ATP (27) using PyMOL software (Schrodinger). Components are colored as follows: CDC123, *green*; ATP, *dark blue*; A109, *red*; and eIF2γ C terminus, *cyan*. *D*, magnification of the CDC123 ATP-binding pocket shown in panel *C*. CDC123-A109 (*red*) and ATP (*dark blue*) are located 3.7 Å apart as indicated. *E*, ribbon representations of human WT CDC123 overlaid with human CDC123-A109V that was generated using AlphaFill (34) and visualized with PyMOL software (Schrodinger). Components are colored as follows: WT CDC123, *green*; CDC123-A109V, *transparent gray*; ATP, *dark blue*; CDC123-A109, *red*; CDC123-A109V, *transparent orange*; WT CDC123-R111, *purple*; CDC123-A109V R111, *transparent pink*. *F*, magnification of WT CDC123 ATP-binding pocket. *Stick* representations of CDC123-A109 (*red*), -R111 (*purple*), and ATP (*dark blue*) are shown. *G*, magnification of CDC123-A109V ATP-binding pocket. Stick representations of CDC123-A109V (*orange*), -R111 (*pink*), and ATP (*dark blue*) are shown. *H*, protein diagram of *Homo sapiens* (*Hs*) CDC123 with the position of A109 indicated in *red* (*top*). Amino acid sequence alignment (*bottom*) of residues surrounding A109 (*red*) in *Hs* CDC123 and its orthologs in the indicated species.

To examine the mechanism of CDC123-mediated eIF2 complex assembly, we utilized rat fibroblasts that homozygously express either wild-type (WT) or a mutant version (A109V) of CDC123 (Fig. S1). CDC123-A109V is a temperature sensitive allele that was previously used in the identification of CDC123 as a key regulator of cell proliferation (20, 21). As protein synthesis and cell division are innately coupled (28, 29, 30, 31, 32, 33), we reasoned that this model would yield insights into eIF2 biogenesis and downstream translational control. Although CDC123-A109V cells grow similarly to their WT counterparts at the permissive temperature (33 °C), A109V caused a significant slow growth phenotype when the cells were placed at the restrictive temperature for up to 48 h (37 °C; Fig. 1, *A* and *B*) (21). A previous crystal structure of fission yeast CDC123 with the budding yeast eIF2γ C terminus (amino acid residues 423–532) and a similar structure containing the human CDC123 with the human eIF2γ C terminus (amino acid residues 362–471) identified CDC123 as an ATP binding protein (23, 27). Analysis of the human CDC123-eIF2γ C-term structure revealed that A109 maps to the CDC123 ATP-binding pocket (Fig. 1, *C* and *D*). To determine how the alanine to valine substitution might impact the CDC123 structure, we used AlphaFold2 with AlphaFill structural prediction software to model the mutant CDC123 protein in complex with ATP (34, 35). We observed similar overall CDC123 protein confirmations in overlays of the human WT CDC123 crystal structure and CDC123-A109V predicted structure in complex with ATP (Fig. 1*E*). However, analysis of the CDC123 ATP-binding pocket revealed striking differences between the WT and mutant proteins (Fig. 1, *F* and *G*). In WT CDC123, the R-group of A109 is located approximately 3.7 Å from the terminal phosphate of ATP, while the nearby positively charged R111 appears to form interactions with the β and γ phosphate groups of ATP (Fig. 1*F*). In contrast, the A109V R-group is 2.7 Å to the β phosphate of ATP, and most likely by steric hindrance, is predicted to alter the confirmation of R111 such that it interacts with the sugar moiety of the ATP molecule (Fig. 1*G*). Of note, the average predicted local distance difference test (pLDDT) scores for the overlaid WT and CDC123 proteins with ATP were approximately 84, while the residues at position 109 and 111 had pLDDTs >90 indicating reliable structural predictions (36). A109 and the surrounding residues in the CDC123 ATP-binding pocket are fully conserved in eukaryotes (Fig. 1*H*). This is an intriguing finding as the CDC123 amino acid sequences assessed from yeast to human only share an overall 25% sequence identity (Fig. S2). Together, these data support the idea that ATP binding and/or hydrolysis are important for CDC123-mediated regulation of cell growth, and highlight A109 as a critical residue for CDC123 function.

### CDC123-A109V impairs eIF2 complex assembly, causing a defect in general mRNA translation

**Figure 2:**
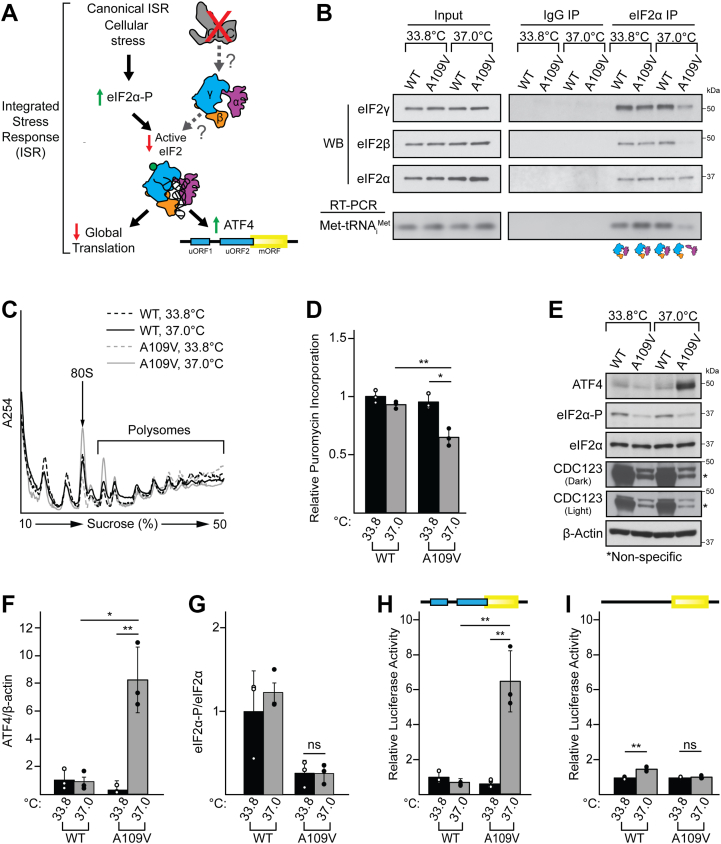
**CDC123-A109V induces downstream readouts of the ISR independent of increased eIF2α-P**. *A*, schematic representation of the canonical, eIF2α-P-mediated ISR alongside the hypothesis tested in this study. Canonical activation of the ISR occurs in response to various cellular stressors (*top left*). In this pathway, stress-sensing eIF2 kinases phosphorylate eIF2 on its α subunit. eIF2α-P inhibits the activity of the eIF2 GEF, eIF2B, resulting in a decrease in the levels of active eIF2 (*i*.*e*., eIF2 bound to both GTP and Met-tRNA_i_^Met^). The two outcomes of decreased active eIF2 are a reduction in global protein synthesis and the translational induction of the stress-responsive factor ATF4 through an upstream open reading frame (uORF)-mediated mechanism. We hypothesized that CDC123-A109V (*gray*, *top right*) would cause a decrease in eIF2 heterotrimer formation, resulting in a downstream reduction in active eIF2 levels and translational readouts of the ISR (*i*.*e*., reduced global protein synthesis and an induction in *ATF4* translation) independent of eIF2α-P. eIF2α, β, and γ subunits are shown in *magenta*, *orange*, and *cyan*, respectively, with GTP indicated by a *green circle*, and Met-tRNA_i_^Met^ in *black*. An *ATF4* 5′-leader schematic is shown with the two uORFs (*cyan boxes*) and the *ATF4* main ORF (*yellow box*; bottom right). *B*, *top* panel: Lysates from WT and CDC123-A109V cells placed at 33.8 °C or 37 °C were subjected to co-immunoprecipitation with eIF2α or control IgG antibodies. Immunoblot analyses (WB) were used to detect the eIF2 subunits in the input and immunoprecipitated (IP) samples. The effect of each test condition on eIF2 heterotrimer formation is indicated below the corresponding lane. eIF2α, β, and γ subunits are shown in *magenta*, *orange*, and *cyan*, respectively. Biological replicate 1 is presented in Figure 2*B*, while biological replicates 2 and 3 are shown in Figure S3, *A* and *B*, respectively. *Bottom* panel: Met-tRNA_i_^Met^ in input and co-immunoprecipitation samples was analyzed by RT-PCR and gel electrophoresis (RT-PCR). Quantification of the WB and RT-PCR images is included in Figure S3*C*. Uncropped images corresponding to this panel are located in Figure S6. *C*, polysome profiles of lysates from WT and CDC123-A109V cells placed at 33.8 °C or 37 °C. Positions of the 80S monosome peak and polysome peaks are indicated. Biological replicate 1 is presented in Figure 2C, while biological replicates 2 and 3 are shown in Figure S4, *A* and *B*, respectively. Average polysome/monosome ratios with error bars (SD) calculated from the 3 biological replicates is shown in Figure S4*C*. *D*, relative puromycin incorporation with error bars (SD) from 40 individual cells randomly selected from three biological replicates. Statistical comparisons between groups were conducted using a one-way ANOVA followed by a post hoc Tukey’s test. Significance is indicated by ∗*p* < 0.05, ∗∗*p* < 0.005. Error bars represent SD from three biological replicates. *E*, protein lysates from WT and CDC123-A109V cells placed at 33.8 °C or 37 °C were subjected to immunoblot analysis for the indicated proteins. Molecular weight markers are shown on the *right*. ∗nonspecific band detected with the CDC123 antibody. Biological replicate 1 is presented in Figure 2*E*, while biological replicates 2 and 3 are shown in Figure S4, *D* and *E*, respectively. Uncropped immunoblots corresponding to this panel are located in Figure S7. *F*, average ATF4/β-actin signal with error bars (SD) from the immunoblots shown in panel *E* and Figure S4, *D* and *E*. Statistical comparisons between groups were conducted using a one-way ANOVA followed by a post hoc Tukey’s test. Significance is indicated by ∗*p* < 0.05, ∗∗*p* < 0.005 (n = 3 biological replicates). Error bars represent SD. *G*, Average eIF2α-P/eIF2α signal with error bars (SD) from the immunoblots shown in panel *E* and Figure S4, *D* and *E*. Statistical comparisons between groups were conducted using a one-way ANOVA followed by a post hoc Tukey’s test. ns = not significant (n = 3 biological replicates). Error bars represent SD. *H*–*I*, WT (*H*) and ΔuORF (*I*) P_TK_-ATF4-Luc constructs were transfected into WT and CDC123-A109V cells placed at 33.8 °C or 37 °C. *ATF4-Luc* translation was measured *via* dual-luciferase assay. Reporter constructs contain the WT or ΔuORF 5′-leader of the *ATF4* mRNA inserted between a minimal TK promoter and the firefly luciferase CDS. When present, uORFs are represented as *blue colored boxes*, and the firefly luciferase CDS is represented as a *yellow box*. In the ΔuORF construct, the uORF start codons have been mutated from AUG to AGG, and the absence of the uORF is denoted by the *lack of blue boxes*. Statistical comparisons between groups were conducted using a one-way ANOVA followed by a post hoc Tukey’s test. Significance is indicated by ∗∗*p* < 0.005, ns = not significant (n = 3 biological replicates). Error bars represent SD. CDS, coding sequence; ISR, integrated stress response; Met-tRNA, methionyl tRNA; TK, thymidine kinase.

During canonical activation of the ISR, phosphorylation of eIF2α reduces the level of active eIF2 by causing a decrease in GTP and Met-tRNA_i_^Met^ binding to the eIF2 heterotrimer (Fig. 2*A*) (1, 11, 37, 38). This reduction in active eIF2 results in two downstream translational consequences: (1) a decrease in global or general mRNA translation and (2) a selective increase in the synthesis of stress-responsive factors, like ATF4 (1, 2, 39). We hypothesized that impaired CDC123 function would cause a significant reduction in eIF2 heterotrimer formation and, subsequently, downstream active eIF2 levels, inducing the translational outcomes of the ISR independent of a canonical increase in eIF2α-P (Fig. 2*A*).

To first assess eIF2 heterotrimer formation in the WT and CDC123-A109V cells, we conducted an eIF2α co-immunoprecipitation (co-IP) followed by immunoblot analysis of the eIF2α, eIF2β, and eIF2γ subunits. Reduced amounts of both eIF2β and eIF2γ were co-immunoprecipitated with eIF2α in lysates from CDC123-A109V cells placed at the restrictive temperature as compared to WT controls grown at 33 °C and 37 °C, or CDC123-A109V cells at 33 °C (Fig. 2*B* and Fig. S3, *A* and *B*; quantification in Fig. S3*C*). In addition, analysis of Met-tRNA_i_^Met^ co-precipitation with eIF2α revealed a significant decrease in active eIF2 levels in CDC123-A109V cells placed at the restrictive temperature compared to the controls (Fig. 2*B* and Fig. S3, *A* and *B*; quantification in Fig. S3*C*). Importantly, in the eIF2 heterotrimer, eIF2α makes direct contact with the eIF2γ subunit, but not with eIF2β (40, 41, 42). CDC123 promotes assembly of the eIF2α-eIF2γ heterodimer, while eIF2β binds to eIF2γ in a CDC123-independent manner (24, 25). Thus, the data in Figure 2*B* indicate that CDC123-A109V impairs CDC123-mediated eIF2 complex assembly at 37 °C through defective binding of eIF2α to eIF2γ, which ultimately decreases levels of the downstream eIF2 TC.

Having confirmed that the CDC123-A109V mutation impairs eIF2 biogenesis, we next tested the second component of our hypothesis: that a reduction in CDC123-mediated eIF2 heterotrimer formation would induce the translational outcomes of the ISR (Fig. 2*A*). In our first assessment of the ISR, we monitored global protein synthesis by polysome profiling of whole cell lysates from WT and CDC123-A109V fibroblasts grown at 33 °C or 37 °C. The polysome profiles revealed a translation initiation defect in CDC123-A109V cells placed at restrictive temperature with a decrease in the heavy polysome fractions and corresponding increase in the 80S monosome and disome fractions as compared to the WT 33 °C, WT 37 °C, and CDC123-A109V 33 °C controls (Fig. 2*C*). Furthermore, with quantification of biological triplicate polysome profiles (Fig. 2*C*, and Fig. S4, *A* and *B*), we observed a significant decrease in the polysome-over-monosome ratio for CDC123-A109V cells grown at the permissive vs. restrictive temperature (Fig. S4*C*, polysome-over-monosome [P/M] ratio decreased from 7.3 ± 0.2 in CDC123-A109V 33 °C to 5.5 ± 0.5 in CDC123-A109V 37 °C). To corroborate these results, overall protein synthesis in WT and CDC123-A109V cells was also assessed by analysis of puromycin incorporation. In line with the polysome profiling data, CDC123-A109V cells placed at the restrictive temperature demonstrated significantly less puromycin incorporation compared to the WT 33 °C, WT 37 °C, and CDC123-A109V 33 °C controls (Fig. 2*D* and Fig. S4*C*). We conclude from these results that CDC123-A109V impaired eIF2 complex assembly, causing a decrease in general translation and a corresponding defect in cell proliferation when the mutant cells were placed at restrictive temperature.

### *ATF4* translation is induced through an eIF2α phosphorylation-independent mechanism in CDC123 mutant cells

As overall protein synthesis was reduced in the CDC123 mutant cells (Fig. 2, *C* and *D*), we next turned our attention to the second translational outcome of the ISR: the preferential synthesis of stress-responsive factors, such as ATF4. Consistent with the idea that the CDC123-A109V mutation induces the ISR at the restrictive temperature, immunoblot analysis revealed an ∼8-fold induction in the expression of ATF4 in CDC123-A109V cells placed at 37 °C (Fig. 2, *E* and *F*, and Fig. S4, *D* and *E*). In line with a previous report, CDC123 protein levels did not change between the CDC123-A109V cells grown at permissive or restrictive temperature (Fig. 2*E*) (21). Importantly, assessment of eIF2α-S51 phosphorylation, the canonical trigger of ISR activation (1), showed no significant changes in eIF2α-P between the CDC123-A109V cells grown at 33 °C versus 37 °C (Fig. 2, *E* and *G*). Furthermore, eIF2α-P levels were markedly lower in the CDC123-A109V cells as compared to their WT counterparts, perhaps due to altered eIF2 kinase or phosphatase activities in these cells. As eIF2α-P levels were not increased in the CDC123-A109V cells placed at restrictive temperature (Fig. 2, *E* and *G*, and Fig. S4, *D* and *E*), the induction in downstream readouts of the ISR likely resulted from reduced CDC123-mediated eIF2 heterotrimer and subsequent TC formation in the CDC123-A109V mutant cells placed at the restrictive temperature (Fig. 2, *A* and *B*).

Previous studies have shown that translational upregulation of *ATF4* during the ISR is controlled by a ribosome reinitiation mechanism that involves two uORFs in the 5′-leader of the *ATF4* mRNA (2, 39, 43). The short, 5′-proximal uORF (uORF1) recruits scanning ribosomes and acts as a positive element by promoting translation reinitiation at downstream ORFs. Under basal (*i*.*e*., no stress) conditions, active eIF2 levels are high, and ribosomes that translate uORF1 resume scanning and are able to quickly reacquire a new eIF2 TC. This promotes ribosome reinitiation at an inhibitory uORF (uORF2) that overlaps out of frame with the main *ATF4* coding sequence (CDS). Translation of uORF2 allows ribosomes to bypass the *ATF4* CDS start codon and results in low ATF4 synthesis under homeostatic conditions. During cellular stress, ribosomes that resume scanning after translation of uORF1 take longer to acquire a new active eIF2 complex as overall levels of the eIF2 TC are reduced by eIF2α-P. Through this mechanism, ribosomes scan past the uORF2 start codon before reacquiring an eIF2 TC and reinitiate translation at the *ATF4* CDS start site. Thus, ATF4 protein synthesis is induced during cellular stress or under conditions in which active eIF2 is limiting (14, 39).

To determine whether ATF4 protein expression was induced *via* the *ATF4* 5′-leader translational mechanism in the CDC123-A109V cells at 37 °C (Fig. 2*E*), we conducted luciferase reporter assays with a construct containing the 5′-leader of *ATF4* mRNA inserted between a minimal thymidine kinase (TK) promoter and the firefly luciferase CDS (P_TK_-ATF4-Luc). CDC123-A109V cells transfected with P_TK_-ATF4-Luc and placed at the restrictive temperature displayed a ∼6-fold increase in luciferase activity compared to the controls (Fig. 2*H*), similar to the level of endogenous ATF4 protein expression observed in these cells (Fig. 2, *E* and *F*, and Fig. S4, *D* and *E*). Mutation of the regulatory uORF1 and two start codons from ATG to AGG in the *ATF4* 5′-leader, effectively removing the uORFs, resulted in constitutive reporter expression in the CDC123-A109V cells grown at both permissive and restrictive temperature (Fig. 2*I*). By comparison, WT cells transfected with the uORF deletion P_TK_-ATF4-Luc construct and placed at their optimal growth temperature of 37 °C (Fig. 1, *A* and *B*) demonstrated a slight increase in luciferase activity as compared to WT cells placed at 33.8 °C (Fig. 2*I*). Combined, these results suggest that the *ATF4* uORF-mediated mechanism directs the temperature dependent translational upregulation of *ATF4* in CDC123-A109V cells. From this, we conclude that the reduction in eIF2 heterotrimer levels in the A109V mutant is sufficient to promote translation of *ATF4* independent of an induction in eIF2α-P.

**Figure 3:**
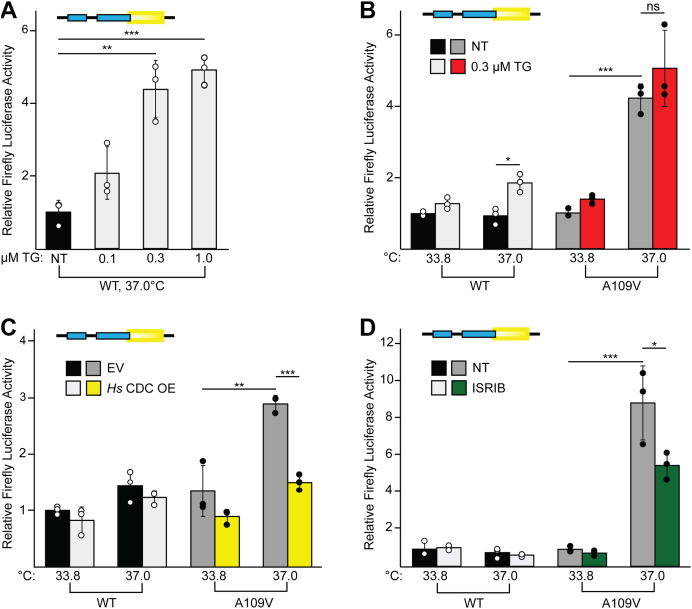
**CDC123-A109V maximally induces ATF4 expression, which can be suppressed by genetic or pharmacological treatments**. *A*, the WT P_TK_-ATF4-Luc construct was transfected into WT cells that were grown at 37 °C and treated with the indicated concentrations of thapsigargin (TG). *ATF4-Luc* translation was measured *via* dual-luciferase assay. Statistical comparisons between groups were conducted using a one-way ANOVA followed by a post hoc Tukey’s test. Significance is indicated by ∗∗*p* < 0.005 (n = 3 biological replicates). Error bars represent SD. *B*, the WT P_TK_-ATF4-Luc construct was transfected into WT, and CDC123-A109V cells placed at 33.8 °C or 37 °C, and *left* untreated (NT) or treated with 0.3 μM thapsigargin (0.3 μM TG). *ATF4-Luc* translation was measured *via* dual-luciferase assay. Statistical comparisons between groups were conducted using a one-way ANOVA followed by a post hoc Tukey’s test. Significance is indicated by ∗*p* < 0.5, ∗∗*p* < 0.005, ∗∗∗*p* < 0.0005, ns = not significant (n = 3 biological replicates). Error bars represent SD. *C*, the WT P_TK_-ATF4-Luc construct was transfected into WT and CDC123-A109V cells placed at 33.8 °C or 37 °C, and co-transfected with an empty vector (EV) or human WT CDC123 overexpression vector (*Hs* CDC OE). *ATF4-Luc* translation was measured *via* dual-luciferase assay. Statistical comparisons between groups were conducted using a one-way ANOVA followed by a post hoc Tukey’s test. Significance is indicated by ∗∗*p* < 0.005, ∗∗∗*p* < 0.0005 (n = 3 biological replicates). Error bars represent SD. *D*, the WT P_TK_-ATF4-Luc construct was transfected into WT and CDC123-A109V cells placed at 33.8 °C or 37 °C, and *left* untreated (NT) or treated with 200 nM ISRIB (ISRIB). *ATF4-Luc* translation was measured *via* dual-luciferase assay. Statistical comparisons between groups were conducted using a one-way ANOVA followed by a post hoc Tukey’s test. Significance is indicated by ∗*p* = 0.05, ∗∗∗*p* < 0.0005 (n = 3 biological replicates). Error bars represent SD. ISRIB, integrated stress response inhibitor; TK, thymidine kinase.

The impacts of the CDC123-A109V mutation on global and gene-specific translation suggest constitutive activation of the ISR. To assess the effect of cellular stress, WT and CDC123-A109V cells were treated with thapsigargin (TG), a potent inducer of ER stress and canonical ISR activation *via* eIF2α-P. We first treated WT cells grown at 37 °C with a range of TG concentrations and found that 0.3 μM TG induced *ATF4* translation to similar levels as cells treatment with a higher TG concentration of 1 μM (Fig. 3*A*). As it was sufficient to induce both eIF2α-P and downstream *ATF4* translation (Figs. S5*A* and 3*A*), we chose to perform subsequent experiments with the lower 0.3 μM TG concentration to avoid potential cytotoxicity associated with the higher 1 μM TG concentration tested. Although induction of the ISR with TG treatment caused an increase in *ATF4* translation in WT cells grown at 37 °C, ATF4 expression was only mildly induced in WT or CDC123-A109V cells grown at the permissive temperature and treated with TG, suggesting that the eIF2 TC is not as limiting for the level of protein synthesis required at this low temperature (Fig. 3*B*). CDC123-A109V cells placed at 37 °C displayed a significant increase in *ATF4* translation basally compared to the WT no treatment control as expected, which was not further enhanced with TG treatment of the CDC123-A109V cells (Fig. 3*B*). Taken together, these data indicate that reduced CDC123-mediated eIF2 complex formation in the mutant cells grown at restrictive temperature is sufficient to maximally induce *ATF4* mRNA translation independent of enhanced eIF2α phosphorylation (Figs. 2*B*, 3*B*, and Fig. S5*A*).

### Basal induction of ATF4 by CDC123-A109V is suppressed by genetic or pharmacological manipulations

In an effort to further elucidate the nature of the CDC123-A109V mutation, we carried out additional P_TK_-ATF4-Luc reporter assays with genetic or pharmacological manipulations of eIF2 levels and activity. First, we overexpressed WT CDC123 in the WT and mutant cells grown at the permissive and restrictive temperatures. As the amino acid motif that surrounds CDC123-A109 is highly conserved between organisms (Fig. 1*H*), we utilized a human WT CDC123 overexpression plasmid for these experiments. Consistent with our previous results, CDC123-A109V cells transfected with P_TK_-ATF4-Luc and an empty vector plasmid displayed a significant induction in ATF4 expression at the restrictive temperature (Fig. 3*C*). Co-transfection of CDC123-A109V 37 °C cells with P_TK_-ATF4-Luc and the human WT CDC123 overexpression vector (*Hs* CDC OE) fully suppressed *ATF4* translation, resulting in similar luciferase activities as the WT control cells (Fig. 3*C*). These data support the conclusion that A109V is a loss-of-function mutation that can be suppressed by WT CDC123.

Notably, the small-molecule integrated stress response inhibitor (ISRIB) was characterized as a suppressor of the ISR that increases eIF2 TC levels by driving GTP and Met-tRNA_i_^Met^ binding to the eIF2 heterotrimer through activation of the eIF2 guanine nucleotide exchange factor eIF2B (44, 45, 46, 47). To assess whether stimulation of eIF2B activity could rescue the phenotypes associated with the CDC123-A109V mutation, we asked whether ISRIB treatment could also suppress ATF4 expression in the mutant cells. As shown in Figure 3*D*, ISRIB treatment partially suppressed the ATF4 induction observed in CDC123-A109V cells grown at restrictive temperature. Together, these data highlight CDC123-A109V as a loss-of-function mutation and suggest that ISRIB-mediated activation of eIF2B can overcome this defect by enhancing GDP to GTP nucleotide exchange on the reduced eIF2 heterotrimer observed in the mutant cells (Fig. 2*B*).

### CDC123-A109V increases ATPase activity, but does not affect ATP binding

**Figure 4:**
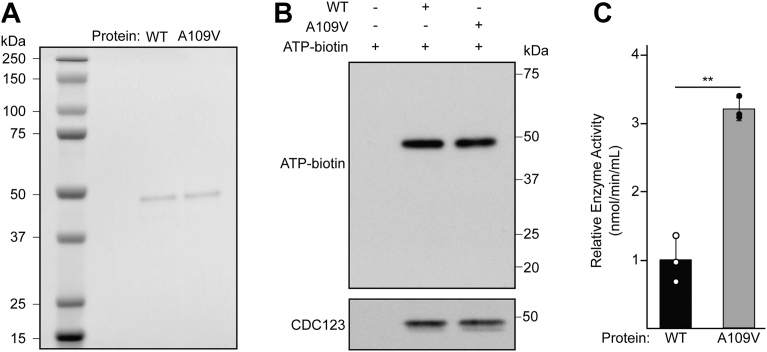
**CDC123-A109V increases spontaneous ATP hydrolysis**. *A*, WT and CDC123-A109V proteins were expressed in and purified from *Escherichia coli*, followed by visualization with Coomassie Blue staining. *B*, purified WT and CDC123-A109V proteins were incubated with a biotinylated ATP analog followed by UV cross-linking. ATP binding to CDC123 was assessed by immunoblot analysis with streptavidin-HRP and a CDC123 antibody. Uncropped immunoblots corresponding to this panel are located in Figure S8. *C*, hydrolysis measurements of ATP bound to purified WT or CDC123-A109V proteins using an ATPase activity kit (Abcam). The average enzyme activity (nmol/min/ml) and error bars (SD) are shown. Statistical comparisons between groups were conducted using an unpaired two-tailed Student’s *t* test. Significance is indicated by ∗∗*p* < 0.005 (n = 3 biological replicates). Error bars represent SD. HRP, horseradish peroxidase.

**Figure 5:**
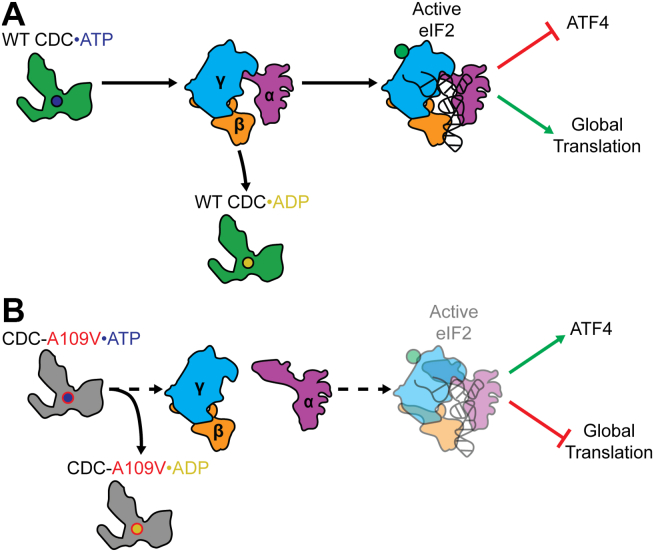
**Spontaneous ATP hydrolysis disrupts CDC123-mediated eIF2 biogenesis and activates the ISR**. *A*, regulated WT CDC123 ATP binding and hydrolysis facilitates eIF2 heterotrimer assembly, suppressing outcomes associated with ISR activation. WT CDC123 is shown in *green* bound to ATP (*blue circle*) or ADP (*yellow circle*). eIF2α, β, and γ subunits are shown in *magenta*, *orange*, and *cyan*, respectively, with GTP indicated by a *green circle*, and Met-tRNA_i_^Met^ in *black*. *B*, we speculate that increased CDC123-A109V ATP hydrolysis is responsible for the observed decrease eIF2 heterotrimer formation, which basally inducing outcomes associated with ISR activation. Mutant CDC123 is shown in *gray* bound to ATP (*red*/*blue circle*) or ADP (*red*/*yellow circle*). eIF2α, β, and γ subunits are shown in *magenta*, *orange*, and *cyan*, respectively, with GTP indicated by a *green circle* and Met-tRNA_i_^Met^ in *black*. ISR, integrated stress response; Met-tRNA, methionyl tRNA.

Having characterized the impacts of the CDC123-A109V mutation on eIF2 biogenesis and translation regulation, we next sought to determine how the mutation impacts CDC123 ATP binding and hydrolysis. After expression and purification of the rat WT and CDC123-A109V proteins from *Escherichia coli*, SDS-PAGE assessment (Fig. 4*A*) revealed a high protein purity of greater than 85 percent. To assess ATP binding to CDC123, we adapted a previously described photo-affinity labeling technique (48). In this procedure, we incubated a biotinylated, nonhydrolyzable ATP analog with either WT or CDC123-A109V proteins at 37 °C for 30 min, followed by UV cross-linking and assessment of ATP binding *via* immunoblot with streptavidin-horseradish peroxidase (HRP) and a CDC123 antibody (Fig. 4*B*). The interaction between the ATP analog and the CDC123 proteins was readily detectible, though, intriguingly, we observed no difference in ATP binding to WT CDC123 or CDC123-A109V (Fig. 4*B*). Importantly, control experiments in which we assessed 2X, 1X, and 0.5X reaction volumes of either WT CDC123 or CDC123-A109V incubated with the ATP analog revealed that the ATP binding experiments were in the linear range of detection (Fig. S5*B*). In addition, no streptavidin-HRP signal was detected from reactions lacking the biotinylated ATP analog, confirming the specificity of the biotin–streptavidin interaction (Fig. S5*C*). In contrast to the ATP-binding experiments, assessment of the ATPase activity of the WT and mutant proteins by measuring the free phosphate generated after incubating the proteins with hydrolyzable ATP at 37 °C for 30 min revealed a 3-fold increase in ATP hydrolysis by CDC123-A109V relative to the WT protein (Fig. 4*C*). Overall, these *in vitro* analyses suggest that increased spontaneous ATP hydrolysis by the CDC123-A109V protein is nonproductive, and support the notion that regulated ATP binding and hydrolysis is critical for CDC123 function (Fig. 5, *A* and *B*).

## Discussion

As noted by the name cell division cycle 123, CDC123 has long been known to be required for cell growth and viability in eukaryotes (19, 21). Studies in budding yeast characterized CDC123 as a protein chaperone required for eIF2 complex formation (22, 25), and we recently determined that the function of CDC123 is conserved in humans (14). Comparative sequence and structural analyses have revealed that CDC123 contains a divergent ATP-grasp fold and can bind to ATP (23, 26, 27). How CDC123 ATP binding and/or hydrolysis contributes to eIF2 complex assembly and translational control, and if this functionality could be targeted from a therapeutic perspective, has remained an intriguing question. Here, we show that mutation of CDC123 in rodent cells results in impaired eIF2 heterotrimer formation, reducing downstream eIF2 TC levels and overall protein synthesis, and providing just our second verification that the function of CDC123 in eIF2 biogenesis is conserved from yeast to mammals. In addition to decreasing general translation, the CDC123-A109V mutation also causes the translational induction of ISR factor ATF4 through uORF-dependent translational control of the *ATF4* mRNA. Although canonical induction of ATF4 during cellular stress is dependent on eIF2α-P, we suggest that the CDC123-A109V mutation, by decreasing eIF2 heterotrimer formation and downstream active eIF2 levels, mimics the effects of eIF2α-P and leads to constitutive activation of the ISR. Moreover, we define CDC123 as an ATPase and find that the CDC123-A109V mutation increases ATP hydrolysis *in vitro*. We hypothesize that this increase in spontaneous CDC123 ATP hydrolysis is nonproductive, potentially leading to alterations in CDC123 binding to or release from the eIF2 subunits (Fig. 5*B*).

### Impaired assembly of the eIF2 complex and activation of the ISR

In support of a link between eIF2 complex assembly and ISR activation, we found here that the CDC123-A109V mutation was sufficient to induce the translational outcomes of the ISR (Fig. 2*A*). Consistent with the temperature sensitive slow growth phenotype of the CDC123-A109V cells (Fig. 1, *A* and *B*) (21), polysome profiling and puromycin incorporation analysis revealed that the mutation caused a significant decrease in general mRNA translation at the restrictive temperature (Fig. 2, *C* and *D*, and Fig. S4, *A* and *B*, *C*). Importantly, this reduction in global protein synthesis and cell proliferation was not driven by an increase in eIF2α-P (Fig. 2, *E* and *G*). Rather, a decrease in CDC123-mediated eIF2 heterotrimer formation, as observed by an impaired association of eIF2α with eIF2γ when the CDC123-A109V cells were placed at 37 °C (Fig. 2*B*, and Fig. S3, *A* and *B*, *C*), was sufficient to suppress overall protein synthesis. These data are also consistent with the known role of CDC123 to specifically facilitate the eIF2α-eIF2γ interaction (22, 23, 25). The defect in eIF2 heterotrimer formation, and downstream eIF2 TC levels, also accounts for the observed elevation in ATF4 translation at the restrictive temperature (Fig. 2, *A* and *B*, *H*). As previously described, the *ATF4* uORF-mediated mechanism of translational control inversely couples the level of active eIF2 complexes with ATF4 protein synthesis (2, 39, 43). Consistent with this idea, overexpression of WT CDC123, which efficiently promotes eIF2 complex assembly and thus readies the eIF2 heterotrimer for GTP and Met-tRNA_i_^Met^ binding, suppressed ATF4 expression in the CDC123-A109V cells (Fig. 3*C*). Similarly, treatment with ISRIB, by driving nucleotide exchange on the diminished level of eIF2 present in the CDC123-A109V cells grown at 37 °C, was sufficient to partially reduce ATF4 expression (Fig. 3*D*). Treatment of the CDC123-A109V cells at restrictive temperature with TG did not further increase ATF4 expression, which was already hyperexpressed as compared to the WT cells (Fig. 3*B*). Based on these data, we would suggest that impaired CDC123 activity, by diminishing the pool of available eIF2 heterotrimer in the cell, appears to decrease the stoichiometric ratio of eIF2:eIF2B to a level that renders eIF2α-P-mediated regulation of eIF2B ineffective in our model. It is interesting then to speculate that the level of eIF2 heterotrimer available in different cell types may contribute to the sensitivity of those cells to eIF2α-P, modulating the level of ISR activation during cellular stress. How CDC123 expression, activity, and downstream eIF2 complex assembly vary between cells and tissues will be critical questions to address in future studies.

Noncanonical induction of the ISR was also recently reported under conditions in which levels of eIF2B are reduced (49). Termed the split-ISR (s-ISR), the authors determined that decreased eIF2B activity in the absence of eIF2α-P results in the translational induction of *ATF4* in an eIF4E-dependent manner that is distinct from the canonical-ISR (c-ISR) mechanism of *ATF4* translational control. Furthermore, through a unique eIF4E-ATF4-PCK2 axis, the s-ISR was described to promote alternative gene expression reprogramming and changes in cellular metabolism, emphasizing the plasticity of the cellular stress response (49). As the eIF2 TC would be expected to be similarly reduced in situations of decreased eIF2B levels or CDC123 activity, it will be interesting to explore if or how impaired CDC123 function contributes to this unique mechanism of modulating cellular homeostasis.

### Therapeutic targeting of the ISR—possibilities for CDC123

Inappropriate regulation of the ISR has been implicated in multiple human diseases, which has led to the development of pharmaceutical strategies that both activate and suppress ISR signaling (11, 50). Previous work has shown that heightened pharmacological induction of the ISR may be beneficial in diseases that are caused by accumulation of misfolded proteins. For example, inhibition of GADD34, the phosphatase regulatory subunit that targets PP1c to dephosphorylate eIF2α-P (51), was found to suppress the molecular and phenotypic defects of the protein misfolding disease amyotrophic lateral sclerosis in mice (52). In this situation, hyperactivation of the ISR *via* the GADD34 inhibitor Sephin1 was thought to reduce the import of newly synthesized proteins into the ER, allowing the ER chaperones and proteasomal machinery to clear the misfolded proteins before accumulating to cytotoxic levels. Similar to ATF4, GADD34 is not constitutively expressed, but is rather translationally induced in response to cellular stressors, including misfolded protein accumulation in amyotrophic lateral sclerosis (2, 10, 53, 54). Thus, the therapeutic benefits of Sephin1 may be limited to situations in which GADD34 expression is sufficiently elevated for GADD34 inhibition to have a meaningful cellular impact. This limitation is an important consideration for diseases like WRS that are caused by reduced eIF2α-P and suppression of the ISR (11, 50). In this scenario, pharmacological induction of the ISR could likely prove beneficial, but GADD34 would not be expected to be expressed (2) or pharmacologically targetable. It is, therefore, important to examine additional mechanisms for pharmacologically inducing the ISR in the treatment of disease.

We propose that targeting CDC123 activity by dysregulating ATP binding or hydrolysis to induce the ISR may be an alternative therapeutic strategy to address diseases caused by ISR hypoactivation. In support of this idea, we determined here that the CDC123-A109V mutation, located in the ATP binding pocket (Fig. 1, *C* and *E*), dysregulates CDC123 activity to induce the ISR (Fig. 5*B*). Comparison of a previously published CDC123 crystal structure to CDC123-A109V AlphaFill structural predictions revealed that the mutation likely causes local conformational changes to the CDC123-ATP binding pocket, moving CDC123-A109V in closer proximity the ATP molecule, and altering the interaction of a nearby arginine residue (R111) with ATP (Fig. 1, *E* and *F*, *G*) (27). Although ATP binding was not significantly impacted by the A109V mutation *in vitro* (Fig. 4*B*, and Fig. S5, *B* and *C*), ATPase activity by CDC123-A109V was strikingly enhanced compared to the WT protein (Fig. 4*C*). We hypothesize that this increase in spontaneous CDC123 ATP hydrolysis is maladaptive and ultimately responsible for the defect in eIF2 heterotrimer formation we observed *in vivo* when the CDC123-A109V cells were placed at their restrictive temperature (Fig. 2*B*, and Fig. S3, *A* and *B*, *C*). However, precisely how CDC123 ATP binding and/or hydrolysis contributes to the formation of critical CDC123-eIF2α and -eIF2γ interactions and/or the release of CDC123 from the eIF2 subunits will be fundamental questions to address in future studies.

Finally, we must also consider the drugability of CDC123. CDC123 belongs to the ATP-grasp superfamily of enzymes that are found in both prokaryotes and eukaryotes, and contain the ATP-grasp fold, an atypical ATP binding site (26, 55). Owing to its distinct features, specific targeting of the ATP-grasp fold in bacterial enzymes has proven fruitful, particularly in the development of ATP-competitive inhibitors (55, 56, 57, 58). These studies, along with ours, lay the groundwork for drug targeting strategies aimed at the human CDC123 ATP-grasp fold. Altogether, our results suggest that modulating CDC123-mediated eIF2 biogenesis by targeting the ATP-binding site could be a therapeutic strategy to induce the ISR in disease scenarios like WRS where eIF2α-P and ISR signaling is suppressed.

## Experimental procedures

### Cell lines, culture conditions, passaging, and freezing

Wild-type (WT; Riken; Cat# RCB0290) and temperature-sensitive mutant (CDC123-A109V; Riken; Cat# RCB0313) rat diploid fibroblast cell lines were maintained in 10 cm dishes at 33.8 °C (permissive temperature) and 5% CO_2_ in Dulbecco’s modified Eagle medium (DMEM) (Thermo Fisher Scientific; Cat# 11965118) supplemented with 10% fetal bovine serum (R&D Systems; Cat# S11150) and 1% penicillin–streptomycin (Gibco; Cat# 15140–122). After reaching 80% confluency, cells were detached with Trypsin-EDTA (Thermo Fisher Scientific; Cat# 25200114) and either subcultured at 33.8 °C or stored in 1 ml freezing media containing DMEM supplemented with 20% fetal bovine serum and 10% dimethyl sulfoxide (Sigma-Aldrich; Cat# D8414) at −80 °C. For individual assays, cells were plated at the specified densities, cultured overnight at 33.8 °C, and either kept at 33.8 °C or shifted to 37.0 °C (restrictive temperature) for the times indicated below.

### Microbe strains

DNA constructs were generated and stored using DH5α *E*. *coli* (New England Biolabs; Cat# C2987H) grown at 37 °C either on LB (Sigma-Aldrich; Cat# L3522) plates or in LB liquid culture with ampicillin (Research Products International; Cat# A40040). Liquid cultures were shaken at 250 RPM.

### Genomic DNA extraction and amplification

WT and CDC123-A109V cells were seeded at 5.5 × 10^6^ cells/well in 10 cm dishes and incubated at 33.8 °C until they reached confluency. Cell lysis was conducted by transferring tissue culture plates to ice, washing cells 2 times with 2 ml 1X PBS, and incubating cells with 750 μl TRIzol LS Reagent on ice for 5 min. Cells were gently detached with a cell scraper, and transferred to microcentrifuge tubes on ice, followed by DNA isolation as described in the TRIzol LS Reagent protocol. REDTaq ReadyMix PCR Reaction Mix was used to amplify a 646-bp region of genomic DNA (that encodes *CDC123*-A109 or -A109V) according to the manufacturer’s protocol. Primers used for amplification were as follows: *CDC123* forward, 5′-GTACTTGGTGACATCTGTGTTTTCAGACATG-3′; *CDC123* reverse, 5′ GTGATGTTTTAAAGAACGCAGACGATGAACAG-3′. For amplification, 10 ng of WT or *CDC123*-A109V genomic DNA template was used in each reaction. The PCR amplicons were subsequently sequenced by Sanger sequencing with a commercial vendor (Azenta Genewiz).

### Cell proliferation assays

WT or CDC123-A109V fibroblasts were seeded at 3750 cells/well in 96-well plates and cultured for 24 h at 33.8 °C. Cells were then placed at either 33.8 °C or 37.0 °C, left to settle for 24 h for the time 0 measurement, or cultured an additional 24 or 48 h. CyQUANT Direct Microplate Reagent (Thermo Fisher Scientific; Cat# C35011) was used to monitor cell proliferation *via* DNA fluorescent labeling according to the manufacturer’s protocol. Briefly, 2X CyQuant Direct Detection Reagent was created by mixing DMEM, CyQUANTDirect nucleic acid stain, and CyQUANT Direct background suppressor. At the time points stated above, 100 μl of 2X CyQUANT Direct Detection Reagent was added to each well, and cells were incubated at 33.8 °C or 37.0 °C for 1 hour. Fluorescence-based quantification of cell proliferation from three biological replicates was conducted using a BioTek Cytation 5 microscope with the fluorescence excitation/emission maxima set to 508/527 nm.

### Immunoblot analyses

After reaching 60% confluency at 33.8 °C, WT or CDC123-A109V cells grown in 10 cm dishes were placed at either 33.8 °C or 37.0 °C for approximately 18 h. To lyse the cells, DMEM was aspirated, and the cells were washed with 5 ml of prechilled 1X PBS per plate. Cells were lysed in 50 μl RIPA buffer (150 mM NaCl (Sigma-Aldrich; Cat# S5150); 5 mM EDTA, pH 8.0 (Invitrogen; Cat# 15575–038); 50 mM Tris–HCl, pH 8.0 (Sigma-Aldrich; Cat# T2694; 1% Triton X-100 (Sigma-Aldrich; Cat# 93443); 0.5% sodium deoxycholate (Sigma-Aldrich Cat# D6750); 0.1% SDS (Sigma-Aldrich; Cat# L3771); 1 cOmplete, EDTA-free protease inhibitor cocktail tablet (Sigma-Aldrich; Cat# 11873580001)) and collected with cell scraping. The lysate was transferred to a 1.5 ml microcentrifuge tube and subsequently sonicated with a Misonix sonicator 3000 for approximately 30 s. Samples were centrifuged for 10 min at 13,000 RPM and 4 °C, and the supernatant was collected into a new microcentrifuge tube on ice. The protein concentration of each lysate was determined using diluted Bio-Rad Protein Assay Dye Reagent Concentrate per the manufacturer’s protocol. Protein samples were normalized, mixed with SDS-sample buffer, and incubated at 70 °C for 10 min. SDS-PAGE was conducted using the Bio-Rad Electrophoresis System and Bio-Rad Criterion TGX Stain-Free 10% polyacrylamide precast gels, followed by protein transfer with a Bio-Rad Criterion Blotter according to the manufacturer’s protocol. Nitrocellulose membranes were blocked in 5% milk in 1X TBS (KD Medical; Cat# RGF-3385) with 0.1% Tween-20 (Promega; Cat# H5152) (TBS-T), followed by incubation with primary antibodies overnight. The following day, membranes were washed three times in TBS-T for 10 min per wash, incubated in the appropriate secondary antibody for 1 h at room temperature, and washed three more times with TBS-T for 10 min each. Immunoblots were developed using Amersham ECL Prime Western Blotting Detection Reagent (Cytiva; Cat# RPN2236) per the manufacturer’s instructions.

Primary and secondary antibodies used in immunoblots for this study are as follows: eIF2α (Bethyl; Cat #: A300–721A), eIF2β (Bethyl; Cat #: A301–742A), CDC123/C10orf7 (GenScript custom rabbit polyclonal antibody), eIF2γ (GenScript custom rabbit monoclonal antibody), ATF4 (Cell Signaling Technologies; Cat #: 11815S), EIF2S1 (phospho S51) (Cell Signaling Technologies; Cat #: ab32157), β-actin (Sigma-Aldrich; Cat #: A5441), Rabbit IgG HRP Linked F(ab′)2 (Cytiva; Cat #: NA9340), and Mouse IgG HRP Linked F(ab′)2 Fragment (Cytiva; Cat #: NA9310). All commercially available antibodies used in this study have been extensively validated by the manufacturer and in the literature. The GenScript CDC123/C10orf7 and eIF2γ primary antibodies were generated against recombinant rat CDC123 and human eIF2γ proteins. The purified primary antibodies were tested for reactivity against their respective recombinant antigen (CDC123 or eIF2γ) and further validated *via* immunoblot and observation of signal intensity at the appropriate molecular weight on rat and human cell lysates. Quantification of all immunoblots was conducted using ImageJ analysis of three independent biological replicates.

### eIF2α co-IP

eIF2α (Bethyl, Cat# A300–721A) or IgG control antibodies (Cell Signaling Technologies; Cat# 2729S) were conjugated to magnetic Dynabeads M270 Epoxy (Thermo Fisher Scientific; Cat#: 14301) as previously described (59) the day prior to cell lysis. Briefly, the antibody was dialyzed in 1X PBS (Quality Biological; Cat# 119–069–131) at 4 °C for 2 h immediately prior to conjugation and was used at a concentration of 10 μg antibody per 1 mg of magnetic beads. During antibody dialysis, 0.75 mg magnetic beads per immunoprecipitation (IP) were transferred to a microcentrifuge tube, washed with 1 ml of 0.1 M sodium phosphate buffer (pH 7.4), vortexed for 30 s, and mixed for 15 min on a tube shaker at room temperature. The microcentrifuge tube was then placed on a magnetic rack, and the supernatant was removed. The magnetic beads were washed a second time with 1 ml of 0.1 M sodium phosphate buffer (pH 7.4), vortexed for 30 s, and the supernatant was removed. The magnetic beads were subsequently resuspended in a reaction mix containing the dialyzed antibody, 1 M ammonium sulfate (ChemCruz; Cat# sc-29187), and 0.1 M sodium phosphate buffer (pH 7.4). Antibodies were conjugated to the magnetic beads overnight at 30 °C with end-over-end rotation. The following day, the microcentrifuge tubes were placed on a magnetic rack, and the supernatant was removed. The beads were then washed sequentially with 1 ml of 0.1 M sodium phosphate buffer (pH 7.4), 1 ml of 100 mM glycine–HCl (ChemCruz; Cat# sc-29187), 1 ml of 10 mM Tris–HCl (pH 8.8), 1 ml of 100 mM triethylamine (Sigma-Aldrich; Cat#: 471283), four 1 ml washes of 1X PBS, 1 ml of PBS with 0.5% Triton X-100 for 15 min, and 1 ml of 1X PBS. The beads were then resuspended in 1 ml of 1X PBS and stored at 4 °C for a maximum of 45 min before incubation with cell lysates.

After reaching 60% confluency at 33.8 °C, 10 cm plates of WT and CDC123-A109V cells were placed at either 33.8 °C or 37.0 °C for approximately 18 h before cell lysis. To lyse the cells, DMEM was aspirated, and the cells were washed with 5 ml of prechilled 1X PBS per plate. Cells were lysed in 200 μl total IP lysis buffer (150 mM NaCl; 50 mM Tris–HCl, pH 8.0; 1% Triton X-100; and 1 cOmplete, EDTA-free protease inhibitor cocktail tablet) and collected with a cell scraper. The lysate was then transferred to a microcentrifuge tube, incubated on ice for 5 min, and cell debris was pelleted by centrifugation at 13,000 RPM for 10 min at 4 °C. The resulting supernatant was collected into a new microcentrifuge tube on ice, and the protein concentration of each lysate was determined using diluted Bio-Rad Protein Assay Dye Reagent Concentrate per the manufacturer’s protocol. For protein input samples, one quarter of the lysate was normalized, mixed with SDS-sample buffer, and incubated at 70 °C for 10 min. For analysis of Met-tRNA_i_^Met^ in input samples, one quarter of the lysate was normalized for protein levels and subjected to RNA isolation using TRIzol LS Reagent. For co-IP experiments, extracts were normalized for protein concentration by diluting the more concentrated extracts with IP lysis buffer to match the protein level of the least concentrated extract in a total volume of 290 μl. One hundred fifteen microliters of each normalized extract was then mixed with 0.75 mg of previously prepared magnetic beads and incubated for 2 h at 4 °C with end-over-end rotation. At a constant temperature of 4 °C, the IP samples were placed on a magnetic rack, and the supernatant was discarded, followed by three washes with 500 μl IP Lysis Buffer. Protein and RNA was eluted by incubating the magnetic beads with either 32 μl 1X SDS Page sample buffer (containing a final concentration of 0.1 M DTT) or 250 μl TRIzol LS Reagent at room temperature for 10 min. Samples incubated with SDS-PAGE sample buffer were then placed on a magnetic rack, and the supernatant was transferred a new tube, and incubated at 70°C for 10 min. Input and co-IP protein samples were subjected to SDS-PAGE and immunoblotting as described above.

RNA was isolated from both the input and co-IP samples incubated with TRIzol LS Reagent following the manufacturer’s protocol. Single-strand complementary DNA (cDNA) synthesis was conducted using the manufacturer’s protocol for SuperScript III First-Strand Synthesis SuperMix and the following Met-tRNA_i_^Met^ specific reverse primer: 5′-GCAGAGGATGGTTTCGATCC-3′. Following cDNA synthesis, PCR reactions with 1 μl of input or co-IP template cDNA were conducted using REDTaq ReadyMix PCR Reaction Mix with 1 μl of forward (5 μM) and reverse primer (5 μM) in a total volume of 25 μl. Primers used in the PCR reactions are as follows: Met-tRNA_i_^Met^ forward, 5′-GCAGAGTGGCGCAGC-3′; and Met-tRNA_i_^Met^ reverse, 5′-GCAGAGGATGGTTTCGATCC-3′. PCR was performed under the following conditions: (1) 94 °C for 2 min; (2) 20 cycles of 94 °C for 30 s; 60 °C for 30 s; and 72 °C for 30 s; and (3) 72 °C for 5 min. Reaction products were resolved by gel electrophoresis on a 2% agarose gel and stained with GelRed Nucleic Acid Stain.

### Polysome profiling by sucrose gradient ultracentrifugation

Polysome profiling was conducted as previously described (60). In brief, WT or CDC123-A109V cells were grown to 30% confluency in 15 cm plates at 33.8 °C and were placed at either 33.8 °C or 37.0 °C for 24 h. Cells were treated with DMEM containing 50 μg/ml of cycloheximide (Sigma-Aldrich; Cat# C7698) for 10 min prior to cell lysis. The plates were transferred to ice, and cells were washed once with 5 ml prechilled 1X PBS containing 50 μg/ml cycloheximide. Cells were lysed in 300 μl passive lysis buffer (20 mM Tris–HCl, pH 7.5; 100 mM NaCl; 10 mM MgCl_2_ (Sigma-Aldrich; Cat# M1028), 0.4% NP-40 (EMD Millipore; Cat# 492016); 50 μg/ml cycloheximide; and 1 cOmplete, EDTA-free protease inhibitor cocktail tablet), detached with a cell scraper, and collected in microcentrifuge tubes placed on ice. The cell lysate was passed through a 1 ml syringe 10 times using a 26-gauge needle, and cell debris was pelleted by centrifugation for 10 min at 13,000 RPM and 4 °C. The concentration of each cell lysate was determined, and lysates were diluted to equal A260 units. One hundred fifty microliters was removed from the top of a 10 to 50% sucrose gradient (20 mM Tris–HCl, pH 7.5; 100 mM NaCl; 10 mM MgCl_2_; 0.4% NP-40; and 50 μg/ml cycloheximide), made using a BioComp Gradient Master, and 300 μl of the cell lysate was gently layered on the top of the gradient. Sucrose gradients were ultracentrifuged using a SW41 Ti rotor at 40,000 RPM and 4 °C for 2 h. Whole cell lysate polysome profiles were collected using a BioComp Gradient Fractionator and a 254-nm UV monitor. Quantification of polysome profiling was completed by calculating the area under the curve of polysomes *versus* monosome of three biological replicates.

### Puromycin incorporation assays

WT or CDC123-A109V cells were seeded at 1 × 10^4^ cells/well in 96-well plates and placed at either 33.8 °C or 37.0 °C for 24 h. The Click-iT Plus OPP Alexa Fluor 488 Protein Synthesis Assay Kit (Thermo Fisher Scientific, Cat# C10456) was used to measure translation levels according to the manufacturer’s protocol. Briefly, cells were incubated in 100 μl 20 μM Click-iT OPP Reagent (Component A) diluted in DMEM and placed at 33.8 °C or 37.0 °C for 30 min. The medium was removed and cells were washed with 100 μl of room temperature 1X PBS. After removing the PBS, cells were fixed with 100 μl per well of 10% Neutral Buffer Formalin and incubated at room temperature for 15 min. The formalin was removed, and cells were permeabilized with 100 μl per well of 0.5% Triton X-100 in 1X PBS, followed by incubation with the Click reaction reagents per the manufacturer’s instructions. Measurements of newly synthesized peptides, in which puromycin had been incorporated, were conducted using a Biotek Cytation 5 microscope with the fluorescence excitation/emission maxima set to 495/519 nm. For quantification, ImageJ (Fiji) was used to determine the average integrated density (fluorescence intensity) from three biological replicates.

### Plasmid constructions

The WT and uORF start codon mutant (ΔuORF) *ATF4* 5′-leader luciferase reporter constructs (P_TK_-ATF4-Luc) used in this study were previously generated and described in (14, 39). In brief, the cDNA segment encoding the *Mus musculus* 5′-leader of *ATF4* was inserted between a constitutive TK promoter and the firefly luciferase CDS in a derivative of plasmid pGL3. The resulting reporter contains the *ATF4* CDS start codon fused in-frame to the firefly luciferase CDS. Site-directed mutagenesis was used to mutate the two uORF start codons in the *ATF4* 5′-leader from ATG to AGG, generating a ΔuORF P_TK_-ATF4-Luc construct. A construct overexpressing WT *Homo sapiens* CDC123 from a pcDNA3.1 vector (P_CMV_- CDC123) was generated by GenScript. All plasmids constructs were sequence verified and maintained in DH5α *E*. *coli* stocks that were stored at −80 °C.

### Plasmid transfections and luciferase assays

WT and CDC123-A109V cells were seeded at 0.45 × 10^6^ cells/well in 6-well plates and incubated at 33.8 °C overnight. Cells were transiently cotransfected with either WT or ΔuORF P_TK_-ATF4-Luc constructs and a *Renilla* control reporter plasmid at a total concentration of 1 μg DNA per well following the Lipofectamine 3000 manufacturer’s instructions. Culture media were exchanged for fresh DMEM with supplements 6 h after transfection and cells were then placed at the permissive (33.8 °C) or restrictive (37.0 °C) for 18 h before lysis. In experiments in which cells were treated with TG, culture media were exchanged 18 h following the first media exchange for culture media either with or without 0.3 μM TG, and cells were placed back at their respective temperatures. Cells were lysed after 6 h of TG treatment (30 h after transfection) following the Dual-Luciferase Reporter Assay System (Promega) protocol. The same procedure was followed for ISRIB treatments except that cells were left untreated or subjected to 200 nM ISRIB treatment. For CDC123 overexpression experiments, cells were transiently co-transfected with either the P_CMV_-CDC123 plasmid or an empty pcDNA3.1 vector control, and both P_TK_-ATF4-Luc and a *Renilla* control reporter plasmid at a total concentration of 1.5 μg DNA per well following the Lipofectamine 3000 manufacturer’s instructions. Cell culture media were exchanged 6 h after transfection, and cells were lysed following the Dual-Luciferase Reporter Assay System protocol. Briefly, 20 μl from each sample was added to one well of a white polystyrene 96-well plate, and luciferase measurements were conducted in a preprogrammed CLARIOstar Plus BMG Labtech Microplate Luminometer. For the firefly luciferase activity measurements, 100 μl of Luciferase Assay Reagent II was added to each well, followed by a 2 s delay, and a 10 s firefly luciferase measurement period. Subsequently, 100 μl of Stop & Glo Reagent was added to each well, followed by a 2 s delay, and a 10 s *Renilla* luciferase measurement period. Quantification of all luciferase assays was conducted by dividing the firefly luciferase activity by the *Renilla* luciferase activity. Biological replicates for each condition were averaged and normalized to the average firefly/*Renilla* luciferase activity calculated for the WT cells grown at 33 °C. Data are presented as the mean ± SD

### ATP binding and hydrolysis assays

His-tagged WT and CDC123-A109V proteins (GenScript custom order) from *Rattus norvegicus* were purified with a Superdex 75 column, followed by a Ni column plus Q column. Purified proteins were stored in 1X PBS and 5% glycerol. Validation of protein purity *via* silver stain by GenScript and our internal validation indicated a protein purity of 85%. Internal validation of protein purity was conducted using Coomassie Brilliant Blue R-250 Staining Solution (Bio-Rad) according to the manufacturer’s protocol.

ATP binding was assessed by photo-affinity labeling of CDC123 proteins with an 8-azido-ATP analog as previously described (48). Briefly, 600 ng of WT or CDC123-A109V protein in 1X PBS buffer with 5% glycerol (pH 7.4) was mixed with 2 μM 8-N3-ATP[γ]biotinpentylamine (Jena Bioscience; Cat# NU-252-BIO) that was resuspended in cross-linking buffer (50 mM Tris–HCl, pH 7.5, 0.1% CHAPS (EMD Millipore; Cat# 220201), 150 mM KCl and 5% glycerol) prior to use. CDC123 and 8-azido-ATP analog reactions were incubated at 37°C with mixing at 300 RPM for 30 min. Samples were then irradiated at 254 nm for 3 min using a UV Stratagene 1800. After cross-linking, samples were mixed with SDS-PAGE sample buffer and DTT for a total reaction volume of 80 μl that was incubated at 70 °C for 5 min. The final protein concentration of the reaction mix was 12 ng/μl. SDS-PAGE was conducted as described in the immunoblot analyses section (see above). Immunoblots were developed using Amersham ECL Prime Western Blotting Detection Reagent (Cytiva) per the manufacturer’s instructions.

ATP hydrolysis was assessed with an ATPase Assay Kit (Abcam; Cat# ab120385) according to the manufacturer’s protocol. Briefly, 600 ng of purified WT or CDC123-A109V proteins were mixed in 96-well microplates with assay buffer that contained 4 mM ATP disodium salt (Abcam; Cat# ab120385) in a total reaction volume of 40 μl. The final concentration of protein per well was 0.3 μM. No protein control wells containing assay buffer, protein storage buffer (1X PBS with 5% glycerol), and ATP were used to correct for background signal. Reaction mixtures were incubated at 37 °C with mixing at 300 RPM for 30 min and absorbance measurements (620 nm) were taken using a CLARIOstar Plus Microplate Reader (BMG Labtech). Enzyme activity (nmol/min/ml) was calculated following the manufacturer’s instructions.

### AlphaFill structural modeling

AlphaFold2 (https://cosmic2.sdsc.edu:8443/gateway) was used to generate monomeric WT and CDC123-A109V protein structural predictions. The files generated from AlphaFold2 were subsequently inputted into AlphaFill (34) (alphafill.eu) to generate WT and CDC123-A109V structural predictions that included ATP. The average pLDDT scores for the WT and CDC123 proteins with ATP were approximately 84. The alanine or valine residues at position 109 and arginine residue at position 111 all had pLDDTs >90. WT and CDC123-A109V protein structures from AlphaFill were visualized and overlaid using PyMOL software (Schrodinger).

### Quantification and statistical analysis

Quantitative data represent the mean ± standard deviation (SD) derived from at least three biological replicates. Statistical significance between test groups was calculated using a unpaired two-tailed Student’s *t* test or one-way ANOVA followed by a post hoc Tukey’s test as indicated in the figure legends. Within the figure legends, ∗ indicates *p* < 0.05, ∗∗ indicates *p* < 0.005, ∗∗∗ indicates *p* < 0.0005. Exact *p* values for all comparisons discussed in the manuscript are indicated in Table S1.

## Data availability

All the experimental data described are included in the manuscript. Raw data are available upon request to Sara Young-Baird (sara.young-baird@usuhs.edu).

## Supporting information

This article contains supporting information.

## Conflict of interest

The authors declare that they have no conflicts of interest with the contents of this article.
