# Supplementary material for: CDC123 is an ATPase that modulates mRNA translation and the integrated stress response by regulating eIF2 complex assembly
Source: J Biol Chem. 2025 Dec 27;302(2):111116. doi: 10.1016/j.jbc.2025.111116 (PMC12860950; doi:10.1016/j.jbc.2025.111116)
Supplement: Supplementary Material [file mmc1.pdf]

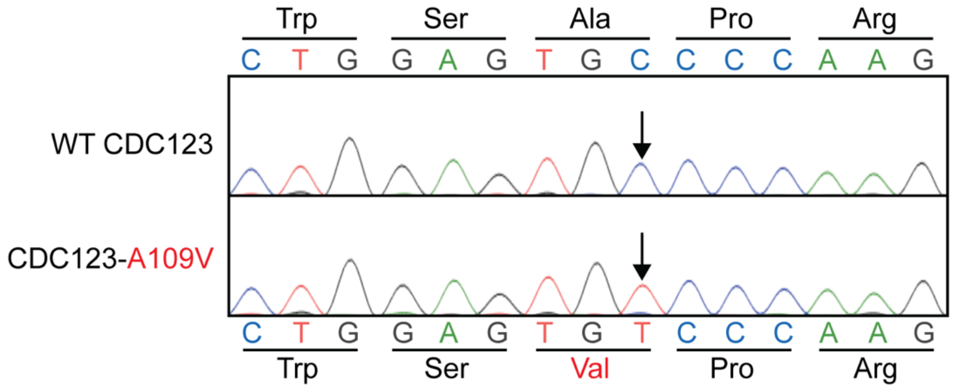

Supplemental Figure 1. Chromosomal *CDC123* sequences from WT and CDC123-A109V cells. *CDC123* sequence chromatograms of the region encoding the CDC123 ATP binding pocket (WT CDC123, top; and CDC123-A109V, bottom). The mutation site, c.379C>T (p.Ala109Val), is indicated by arrows.

```
H.sapiens          -------------MKKEHVLHCQFSAWYPFFRGVTIKSVILP-LPQNVKDYLLDDGTLVV
R.norvegicus       -------------MKKEHVSHCQFSAWYPLFRSLTIKSVILP-LPQNVKDYLLDDGTLVV
M.musculus         -------------MKKEHVSHCQFSAWYPLFRSLTIKSVILP-LPQNVKDYLLDDGTLVV
S.scrofa           -------------MKKEHVLHCQFSAWYPLFRSLTIKSVILP-LPQNVKDYLLDDGTLVV
G.gallus           -------------MKKEQVAHCQFSVWYPLFRAVTIRSVILP-LPENVKEYLLDDGTLVV
D.rerio            -------------MKKEQVVNCQFSVWYPLFKKHTIKSLILP-IPQNVIDYLLDDGTLVV
S.cerevisiae       MSSQEYTTFIDIPVTRAQVEHCSYSFWSSLYPKYVPKSIVLKSLPKKFIQYLEQDGIKLP
                                :.: :* :*.:* * .::   . :*::*  :*::. :** :**  :

H.sapiens          SGRDDPPTHSQPD-SDDEAEEIQWSDDENTATLTAP------EFPEFATKVQEAINSLGG
R.norvegicus       SGREDPPTCSQPD-SGDEAEETQWSDDESTATLTAP------EFPEFNTQVQEAINSLGG
M.musculus         SGREDPPTCSQSD-SGNEAEETQWSDDESTATLTAP------EFPEFNTQVQEAINSLGG
S.scrofa           SGREDPPARSQPD-SDDEAEEIQWSDDENTATLTAP------EFPEFTTKVQEAINSLGG
G.gallus           SGREDPPTQTPEG-SDD-AEEIQWSDDENTATLKAP------EFPEFTAKVEEAISSLGG
D.rerio            SGSENNNSQTQANNSDSDEEDIQWTDDETTTTVTAP------EFPEFNVKVQEAINVLGG
S.cerevisiae       QEENSRSVYTEEIIRNEDNDYSDWEDDEDTATEFVQEVEPLIDFPELHQKLKDALNELG-
                   .  :.    :     ..  :  :* *** *:*  .       :***:  ::::*:. **

H.sapiens          SVFPKLNWSAPRDAYWIAMNSSLKCKTLSDIFLLFKSSDFITRDFTQPFIHCTDDSPDPC
R.norvegicus       SVFPKLNWSAPRDAYWIAMNSSLKCKSLSDIFLLFKSSDFITHDFTQPFIHCNDDSPDPC
M.musculus         SVFPKLNWSAPRDAYWIAMNSSLKCKTLSDIFLLFKSSDFITHDFTQPFIHCTDDSPDPC
S.scrofa           SVFPKLNWSAPRDAYWIAMNSSLKCKTLSDIFLLFKSSDFITRDFTQPFIHCTDDSPDPC
G.gallus           SVFPKLNWSAPRDAYWIAMNSSLKCKALSDIFLLFKSSDFITRDLTQPFIHCTDDSPDPS
D.rerio            CIFPKLNWSAPRDANWIALNSSLQCQSLSEIFLLFKSSDFITHDLTQPFLHCSDDSPDPT
S.cerevisiae       AVAPKLNWSAPRDATWILPNNTMKCNEVNELYLLLNASNYIMHDLQRAFKGCVDGDDIKG
                   .: *********** **  *.:::*: :.:::**:::*::* :*: :.*  * *..

H.sapiens          IEYELVLRKWCELIPGAEFRCFVKENKLIGISQRDYTQYYDHISKQKEEIRRCIQDFFKK
R.norvegicus       IEYELVLRKWCELIPGAEFRCFVKENKLIGISQRDYTQYYDHISKQKEEICRCIQDFFKE
M.musculus         IEYELVLRKWCELIPGAEFRCFVKENKLIGISQRDYTQYYDHISKQKEEICRCIQDFFKE
S.scrofa           MEYELVLRKWCELIPGAEFRCFVKENKLIGISQRDYTQYYDHISKQKEEICRCIQDFFKK
G.gallus           LNYELVLRKWCELIPGAEFRCFVKENKLIGISQRDYTQYYDHISKQHEEICRSIQEFFKK
D.rerio            INYELVLRKWSELIPGGEFRCFVKENKLIAICQRDYTQHYQHIGKQEASISTSILQFFRD
S.cerevisiae       LKFDLVLRQWCDMNPALEFRVFVKNAHIVGATQRDLN-YYDYLDELSDTFKDLIDEIVHD
                   ::::****:*.:: *. *** ***: :::.  *** . :*:::.:    :   * ::.:.

H.sapiens          HIQYKFLDEDFVFDIYRD-SRGKVWLIDFNPFGEVTDSLLFTWEELISENNLNGDFSEVD
R.norvegicus       HLQYKFLDEDFVFDIYRD-SRGKVWLIDFNPFGEVTDSLLFTWEELTSENNLRGDVSEAD
M.musculus         HLQYKFLDEDFVFDIYRD-SRGKVWLIDFNPFGEVTDSLLFTWEELTSENNLRGEVTEGD
S.scrofa           HIQYKFLDEDFVFDIYRD-SRGKVWLIDFNPFGEVTDALLFTWEELLSGRSVRGDLSEGE
G.gallus           HIQYKFLDEDFVFDVYRD-SRGKIWLIDFNPFGEVTDSLLFTWDELTSGRNLKGDQSEVE
D.rerio            NIQYQFPDEDFVLDVYRD-SSGRVWLIDFNPFGEVTDSLLFTWEELTSGKNLTANQTQEE
S.cerevisiae       VVLPKFPDKSFVLDVYIPRPFNKIFIVDINPFARKTDSLLFSWNEIAAIAPPKNDVEDYE
                    :  :* *:.**:*:*   . .:::::*:***.. **:***:*:*: :      : :  :

H.sapiens          AQEQDSPAFRCTNSEVTVQPSPYLSYRLPKDFVDLSTG-EDAHKLIDFLKLKRNQQEDD-
R.norvegicus       ALEQDSPAFRCTNSEVTVQPSPYLSYGLPKDFVDLSTG-EDAHKLIDFLKLKRNQQEDD-
M.musculus         AQEQDSPAFRCTNSEVTVQPSPYLSFGLPKDFVDLSTG-EDAHKLIDFLKLKRNEQEDD-
S.scrofa           APEQDAPTFRCTNSEVTVQPSPYLSYRLPKDFVDLSTG-EDAHKLIDFLKLKRNQQEDD-
G.gallus           ATEQDYPVFRCXNSQVTVQPSPYLSYRLPKDFVDLSTG-EDVHKLIDFLKLKRNQQDDD-
D.rerio            TALPDGPAFRCTNSEVTVQPSPCLSYRIPRDFLDLTTG-EDAYKLIDFLKLKRGQQEEEE
S.cerevisiae       LR------LVTRHNTGRFASKEHSENHVPQDLVEASLNPEAIRELTQKWKELLSQQAKEE
                           :   :.   . ..   .  :*:*::: : . *   :* :  *   .:* .:

H.sapiens          ----------
R.norvegicus       ----------
M.musculus         ----------
S.scrofa           ----------
G.gallus           ----------
D.rerio            ESNEEGEEPQ
S.cerevisiae       SSDSENET--
```

Supplemental Figure 2. Multiple sequence alignment of CDC123 amino acid sequences from *H. sapiens*, *R. norvegicus*, *M. musculus*, *S. scrofa*, *G. gallus*, *D. rerio*, and *S. cerevisiae.* A109 (red, bolded) is located within a highly conserved amino acid motif (black, bolded). Fully conserved, strongly similar, weakly similar, and non-conserved residues are indicated by an asterisk (*), semicolon (:), period (.), and no symbol, respectively.

A

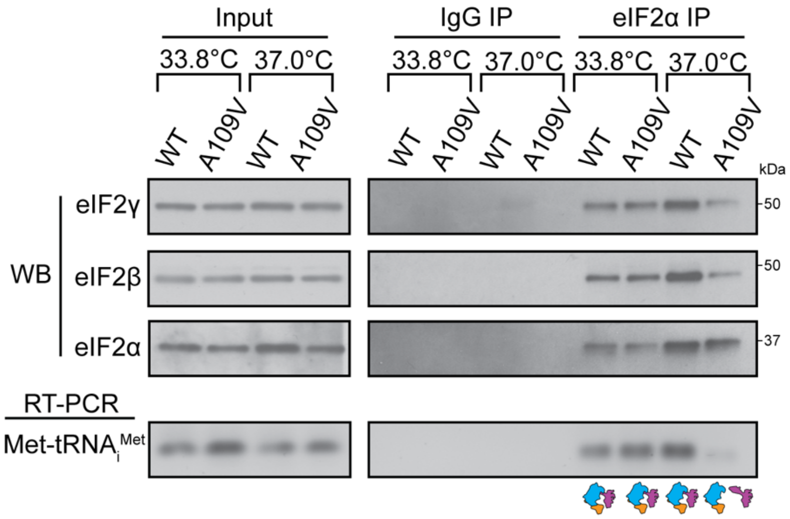

B

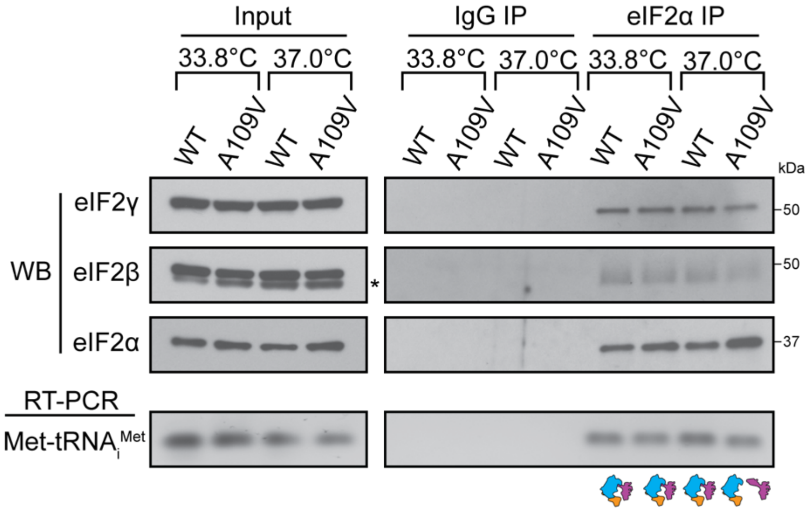

C Immunoprecipitation Quantification

Normalized Comparison	33.8°C WT	33.8°C A109V	37.0°C WT	37.0°C A109V
eIF2β/eIF2α	1.00 ± 0.00	0.79 ± 0.21	0.95 ± 0.13	0.25 ± 0.12
eIF2γ/eIF2α	1.00 ± 0.00	0.89 ± 0.38	0.94 ± 0.17	0.33 ± 0.03
Met-tRNA$_i^{Met}$/eIF2α	1.00 ± 0.00	1.04 ± 0.40	0.98 ± 0.10	0.29 ± 0.16

Supplemental Figure 3. CDC123-A109V disrupts CDC123-mediated eIF2 complex assembly – related to Figure 2B.

(A-B) Lysates from WT and CDC123-A109V cells placed at 33.8°C or 37°C were subjected to co-immunoprecipitation with eIF2α or control IgG antibodies. Immunoblot analyses were used to detect the eIF2 subunits in the input and immunoprecipitated (IP) samples. The effect of each test condition on eIF2 heterotrimer formation is indicated below the corresponding lane. eIF2α, β, and γ subunits are shown in magenta, orange, and cyan, respectively. Biological replicate 1 is presented in Figure 2B, while biological replicates 2 and 3 are shown in Supplemental Figure 3A and 3B, respectively. Met-$tRNA_i^{Met}$ in input and co-immunoprecipitation samples was also analyzed by RT-PCR and gel electrophoresis; western blot (WB) and RT-PCR panels are indicated. Uncropped immunoblots corresponding to panels A and B are located in Supplemental Figures 9 and 10, respectively.

(C) Quantification of immunoblots and RT-PCR from Figure 2B, and Supplemental Figure 3A and 3B. Values shown are the average signal intensity and SD from three biological replicates. These values have been normalized to the WT cells placed at the permissive temperature (33.8°C).

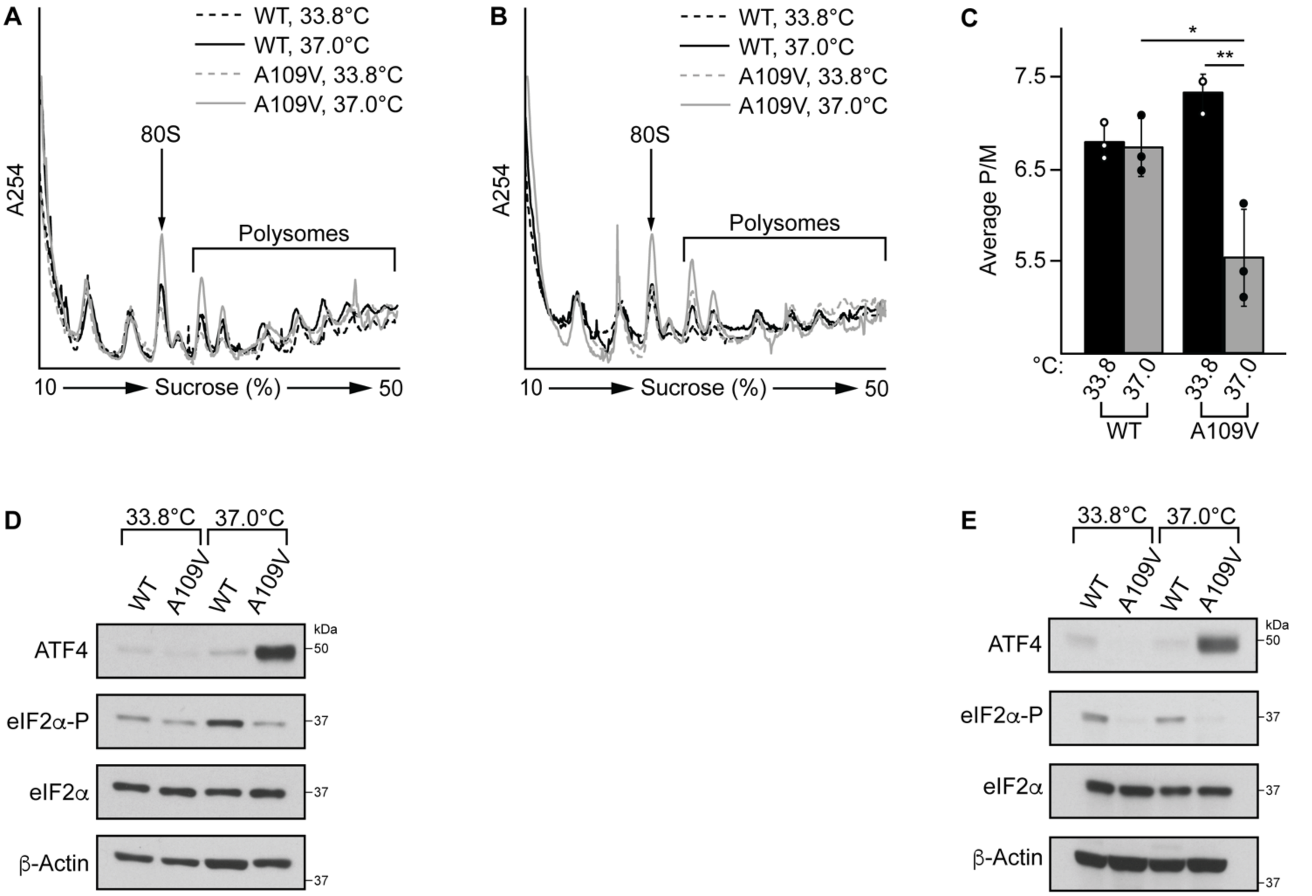

Supplemental Figure 4. CDC123-A109V suppresses general protein synthesis while inducing expression of ATF4.

(A-B) Polysome profiles of lysates from WT and CDC123-A109V cells grown at 33.8°C or 37°C. Positions of 80S monosome and polysomes are indicated. Biological replicate 1 is presented in Figure 2C, while biological replicates 2 and 3 are shown in Supplemental Figure 4A and B, respectively.

(C) Average polysome/monosome ratios with error bars (SD) calculated from the replicates shown in Figure 2C and Supplemental Figures 4A and 4B. Statistical comparisons between groups were conducted using a 1-way ANOVA followed by a post-hoc Tukey's test, significance is indicated by *$p<0.05$, **$p<0.005$ (n=3 biological replicates). Error bars represent SD.

(D-E) Protein lysates from WT and CDC123-A109V cells placed at 33.8°C or 37°C were subjected to immunoblot analysis for the indicated proteins. Molecular weight markers are shown on the right. Biological replicate 1 is presented in Figure 2E, while biological replicates 2 and 3 are shown in Supplemental Figures 4D and 4E, respectively. Uncropped immunoblots corresponding to panels D and E are located in Supplemental Figures 11 and 12, respectively.

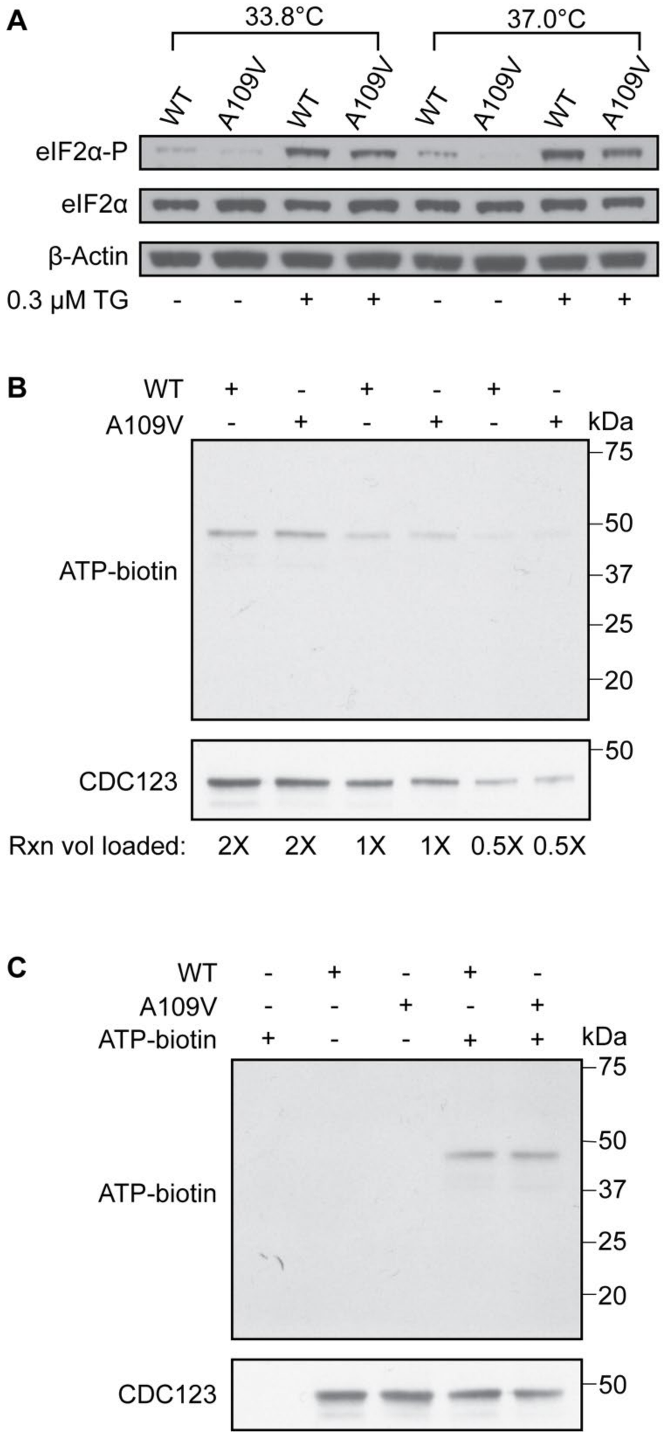

Supplemental Figure 5. CDC123-A109V does not impact ATP binding.

(A) Protein lysates from WT and CDC123-A109V cells placed at 33.8°C or 37°C, and left untreated (NT) or treated with 0.3 μM thapsigargin (0.3 μM TG), were subjected to immunoblot

analysis for the indicated proteins. Uncropped immunoblots corresponding to panel A are located in Supplemental Figure 13.

(B) Purified WT and CDC123-A109V proteins were incubated with a biotinylated ATP analog followed by UV crosslinking. Reaction volumes representing 2X, 1X, and 0.5X were loaded onto the gel to assess the linear range of detection. ATP binding and CDC123 levels were assessed by immunoblot analysis with streptavidin-HRP and a CDC123 antibody. Uncropped immunoblots corresponding to panel B are located in Supplemental Figure 14.

(C) Purified WT and CDC123-A109V proteins were incubated with or without a biotinylated ATP analog followed by UV crosslinking. ATP binding and CDC123 levels were assessed by immunoblot analysis with streptavidin-HRP and a CDC123 antibody. Uncropped immunoblots corresponding to panel C are located in Supplemental Figure 15.

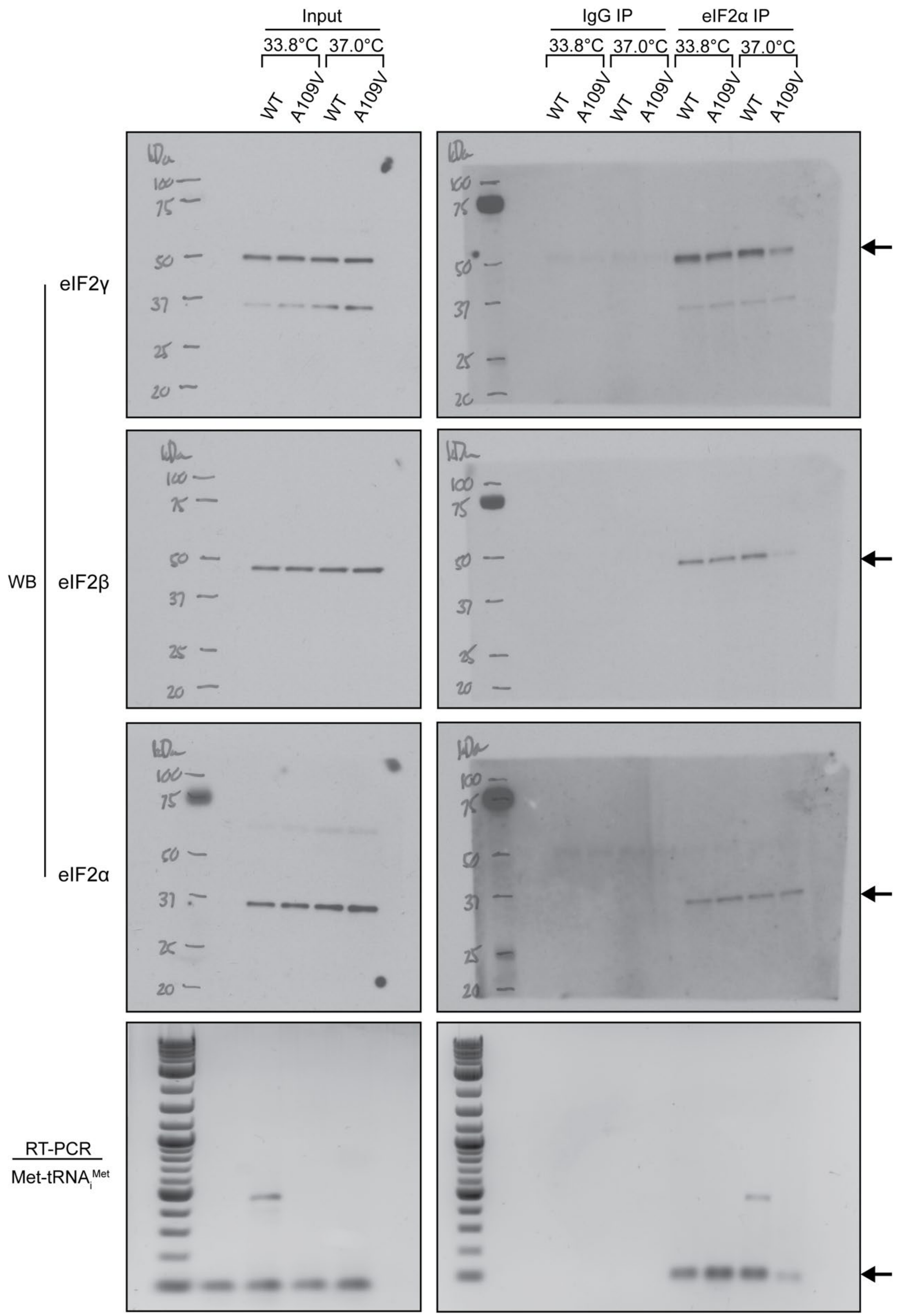

Input
33.8°C
37.0°C
WT
A109V
WT
A109V
IgG IP
eIF2α IP
33.8°C
37.0°C
33.8°C
37.0°C
WT
A109V
WT
A109V
WT
A109V
WT
A109V
WB
eIF2γ
eIF2β
eIF2α
RT-PCR
Met-tRNAiMet

Supplemental Figure 6. Uncropped immunoblot images to accompany Figure 2B.

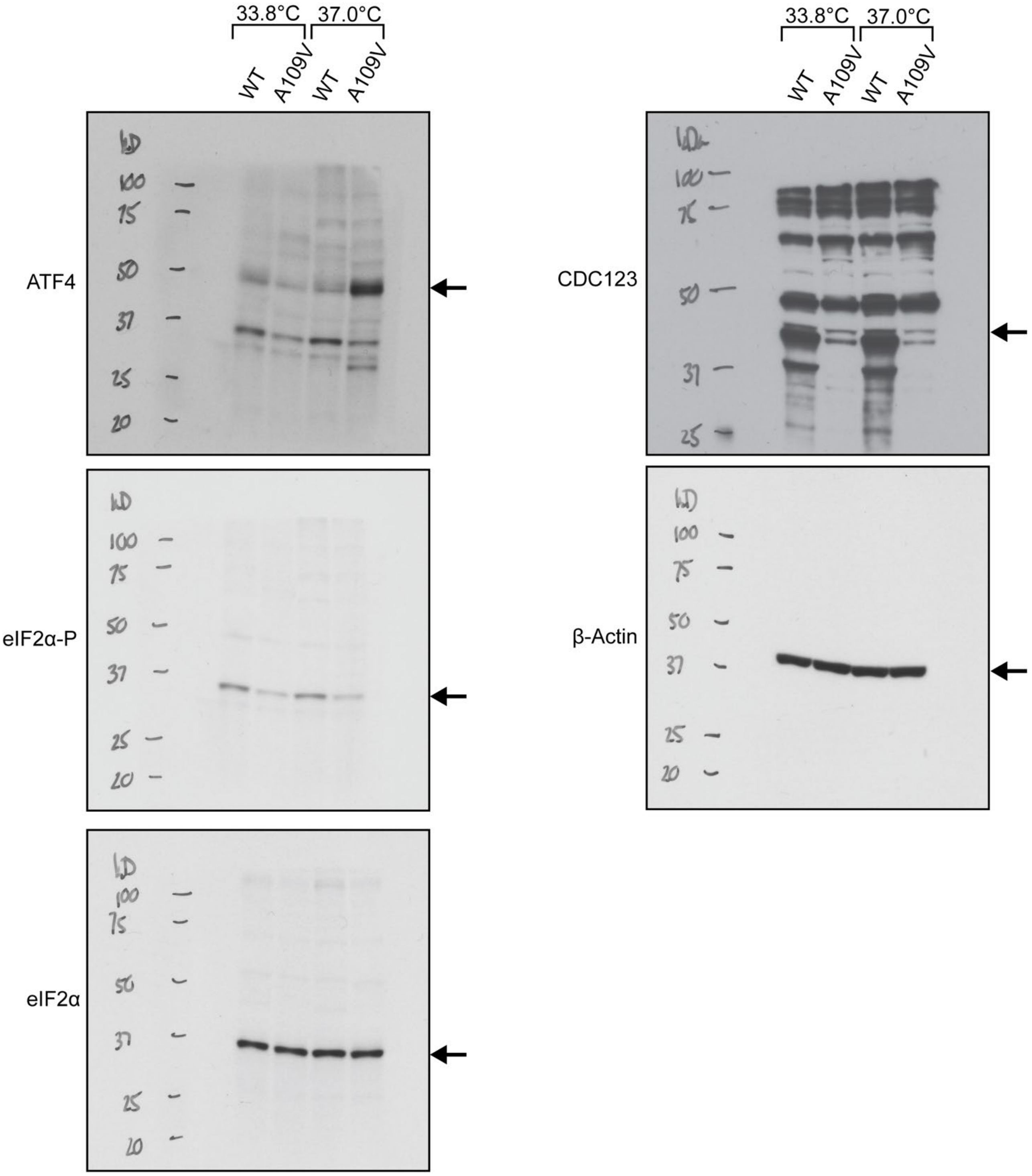

Supplemental Figure 7. Uncropped immunoblot images to accompany Figure 2E.

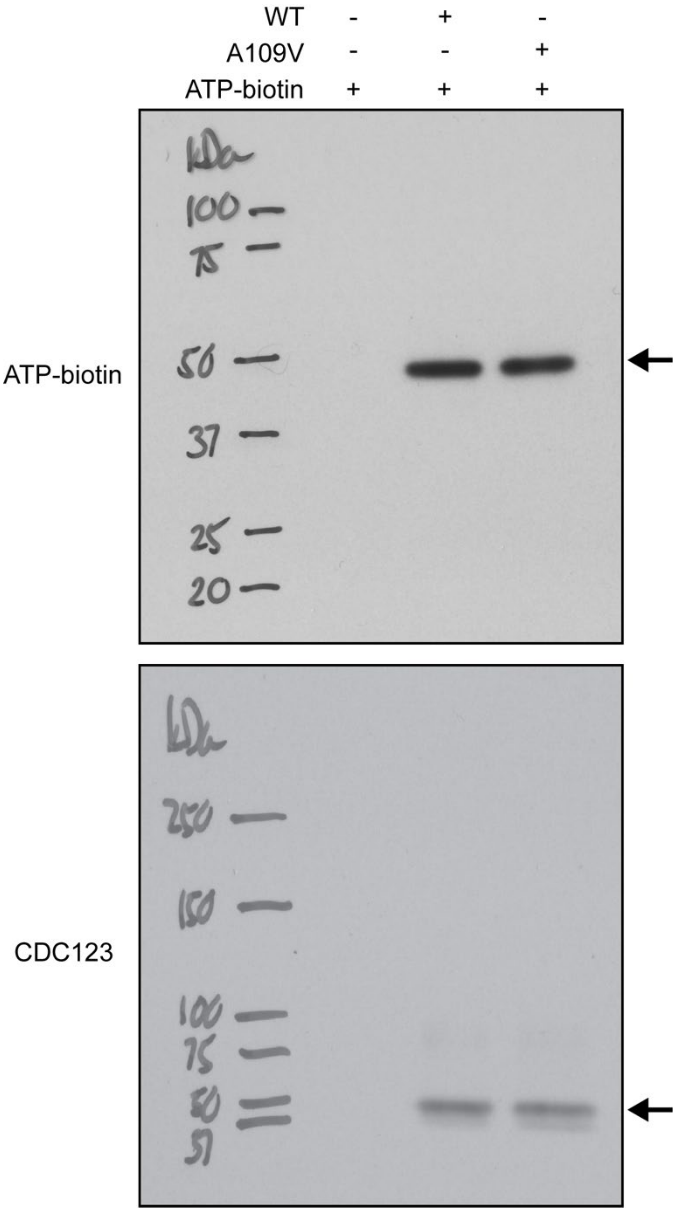

Supplemental Figure 8. Uncropped immunoblot images to accompany Figure 4B.

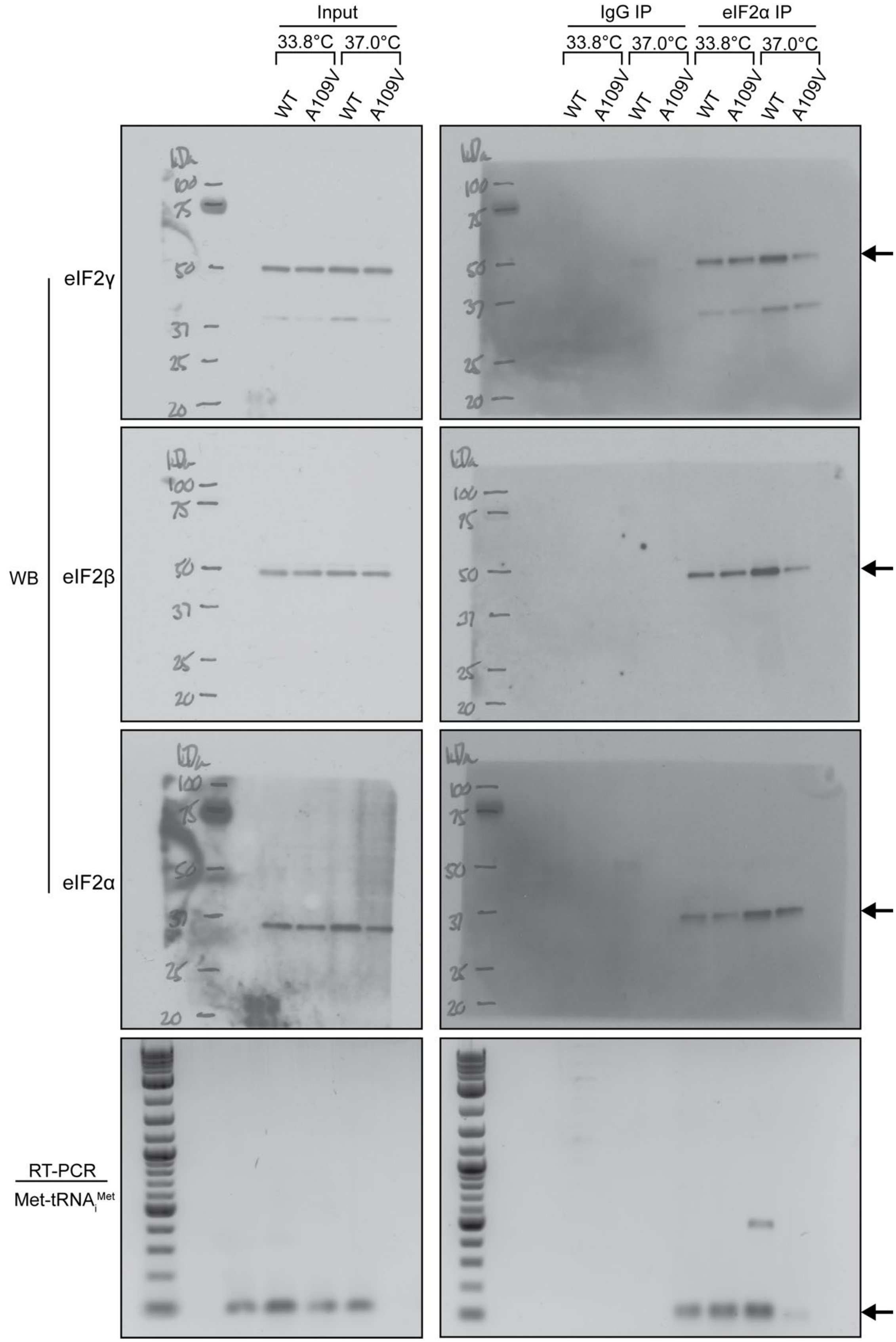

Input
33.8°C
37.0°C
WT
A109V
WT
A109V
IgG IP
eIF2α IP
33.8°C
37.0°C
33.8°C
37.0°C
WT
A109V
WT
A109V
WT
A109V
WT
A109V
WB
eIF2γ
eIF2β
eIF2α
RT-PCR
Met-tRNAi Met
kDa
100
75
50
37
25
20

Supplemental Figure 9. Uncropped immunoblot images to accompany Supplemental Figure 3A.

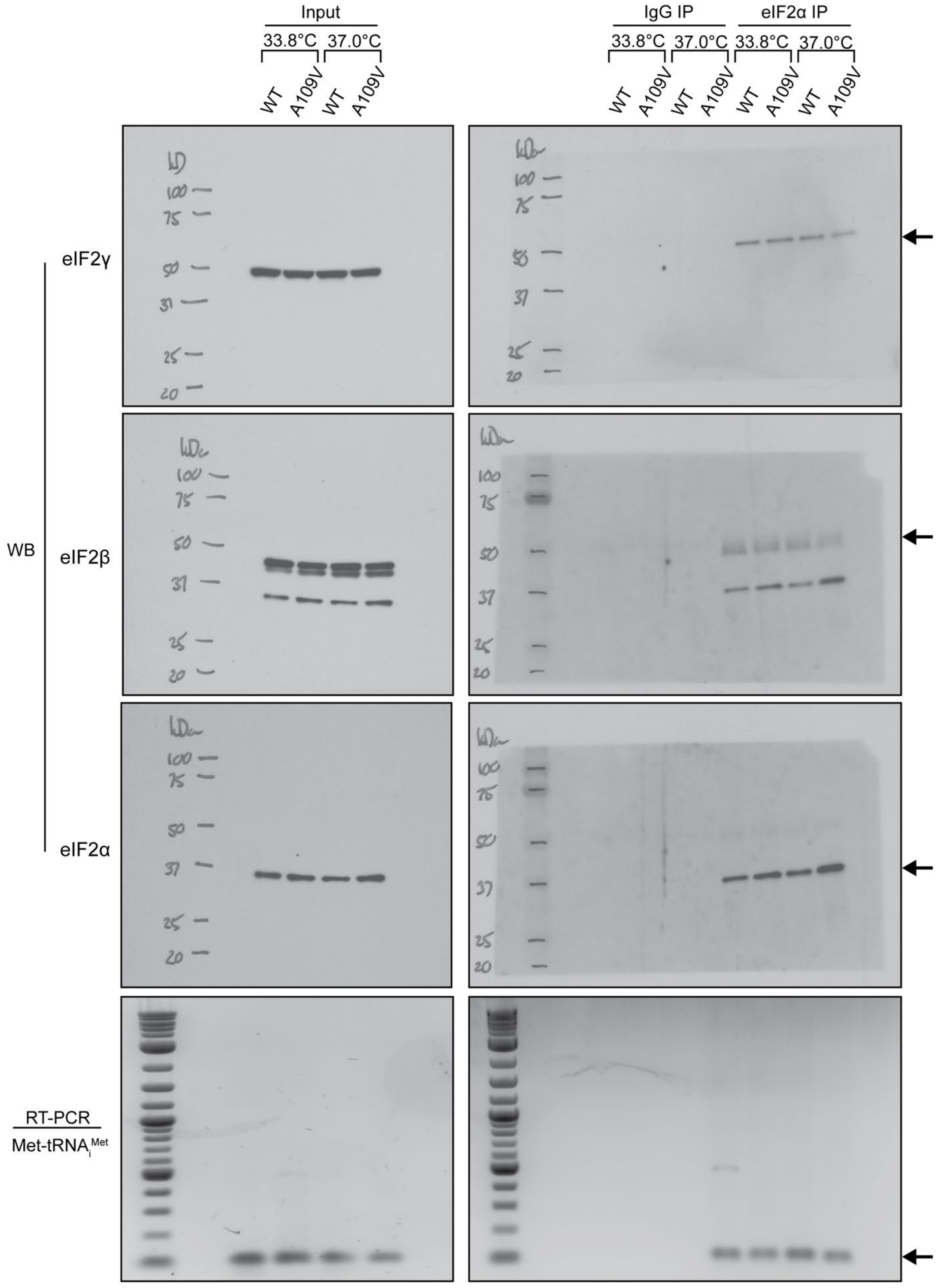

Input
33.8°C
37.0°C
WT
A109V
WT
A109V
IgG IP
eIF2α IP
33.8°C
37.0°C
33.8°C
37.0°C
WT
A109V
WT
A109V
WT
A109V
WT
A109V
WB
eIF2γ
eIF2β
eIF2α
RT-PCR
Met-tRNAi Met

Supplemental Figure 10. Uncropped immunoblot images to accompany Supplemental Figure 3B.

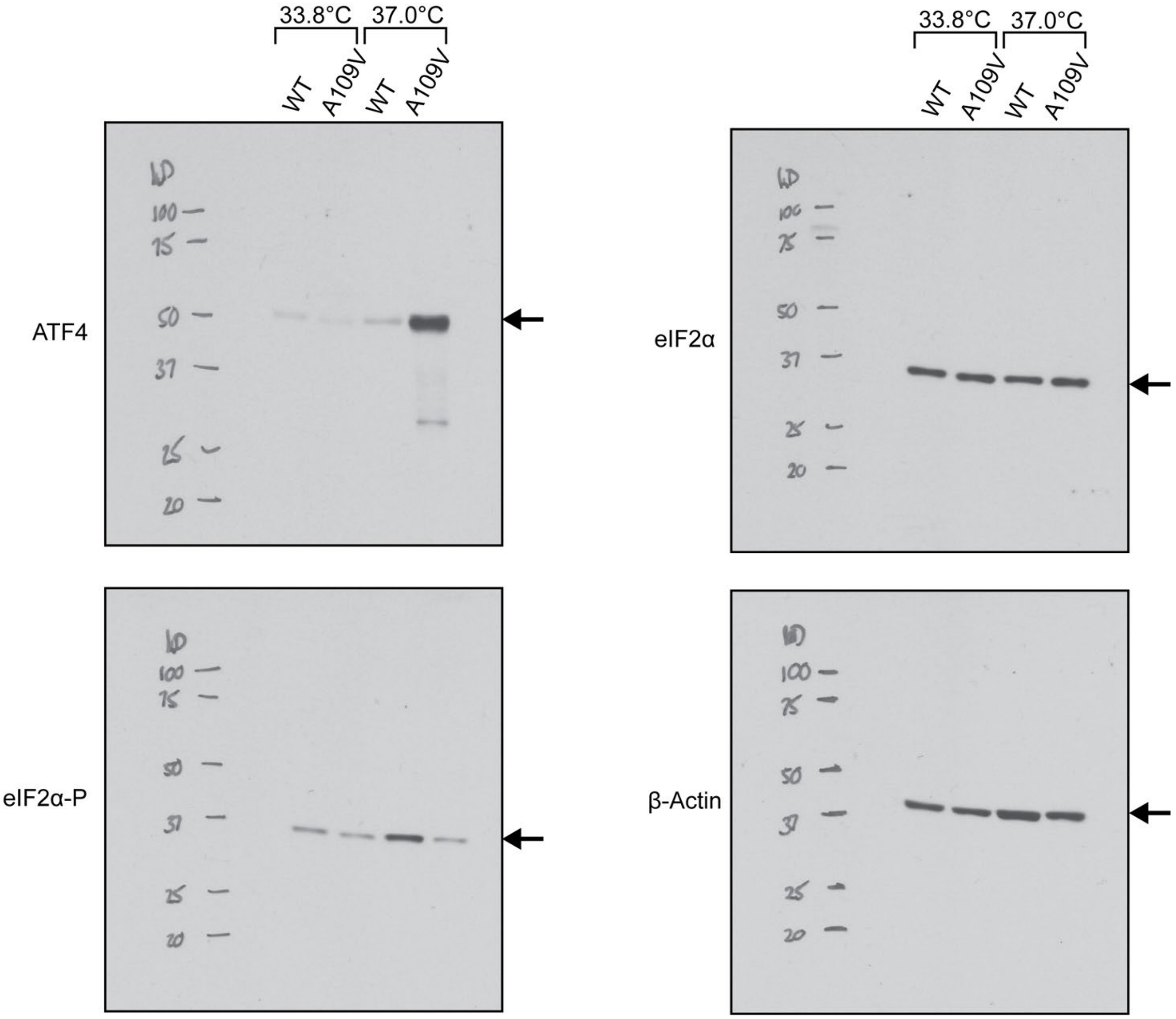

Supplemental Figure 11. Uncropped immunoblot images to accompany Supplemental Figure 4D.

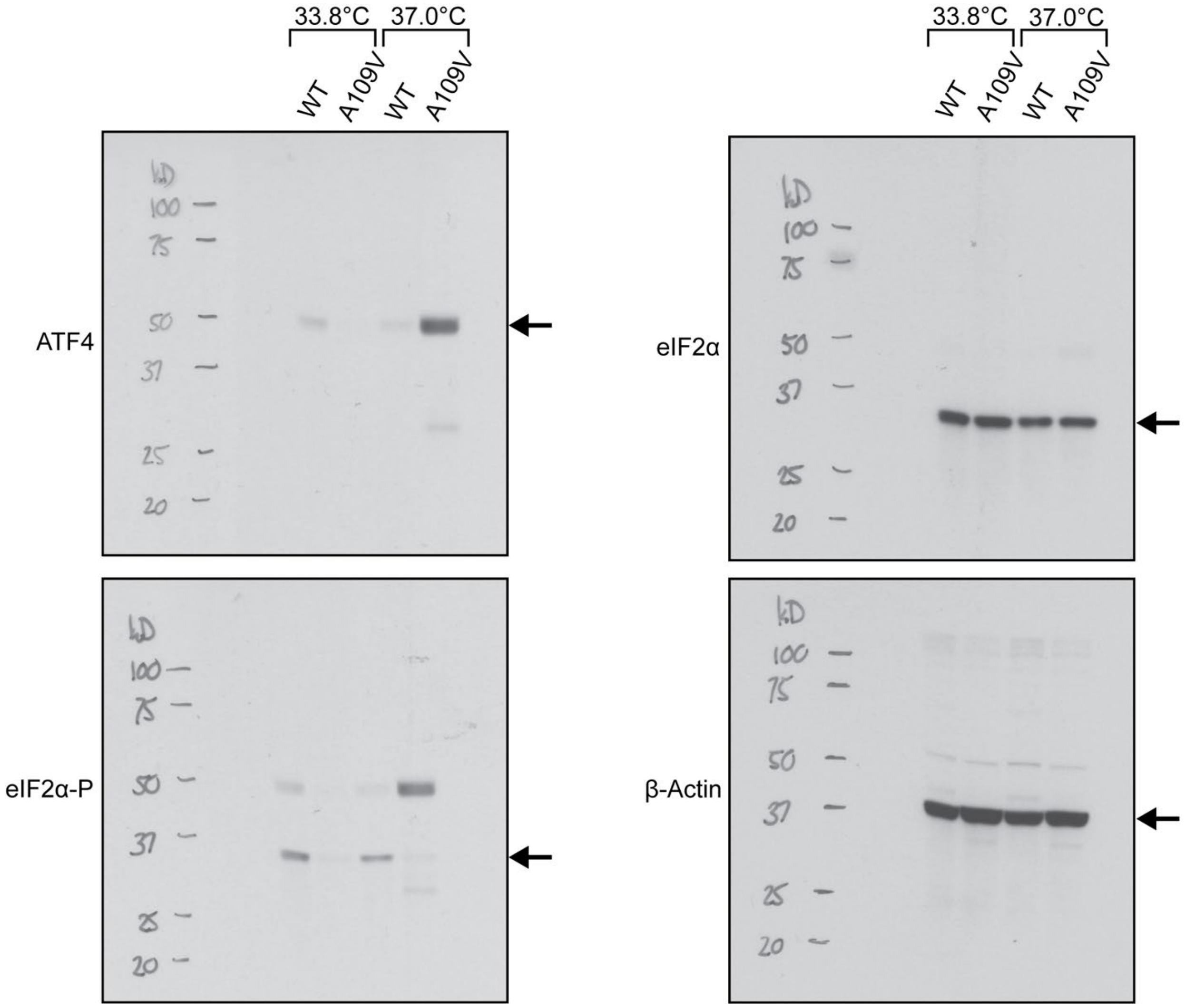

Supplemental Figure 12. Uncropped immunoblot images to accompany Supplemental Figure 4E.

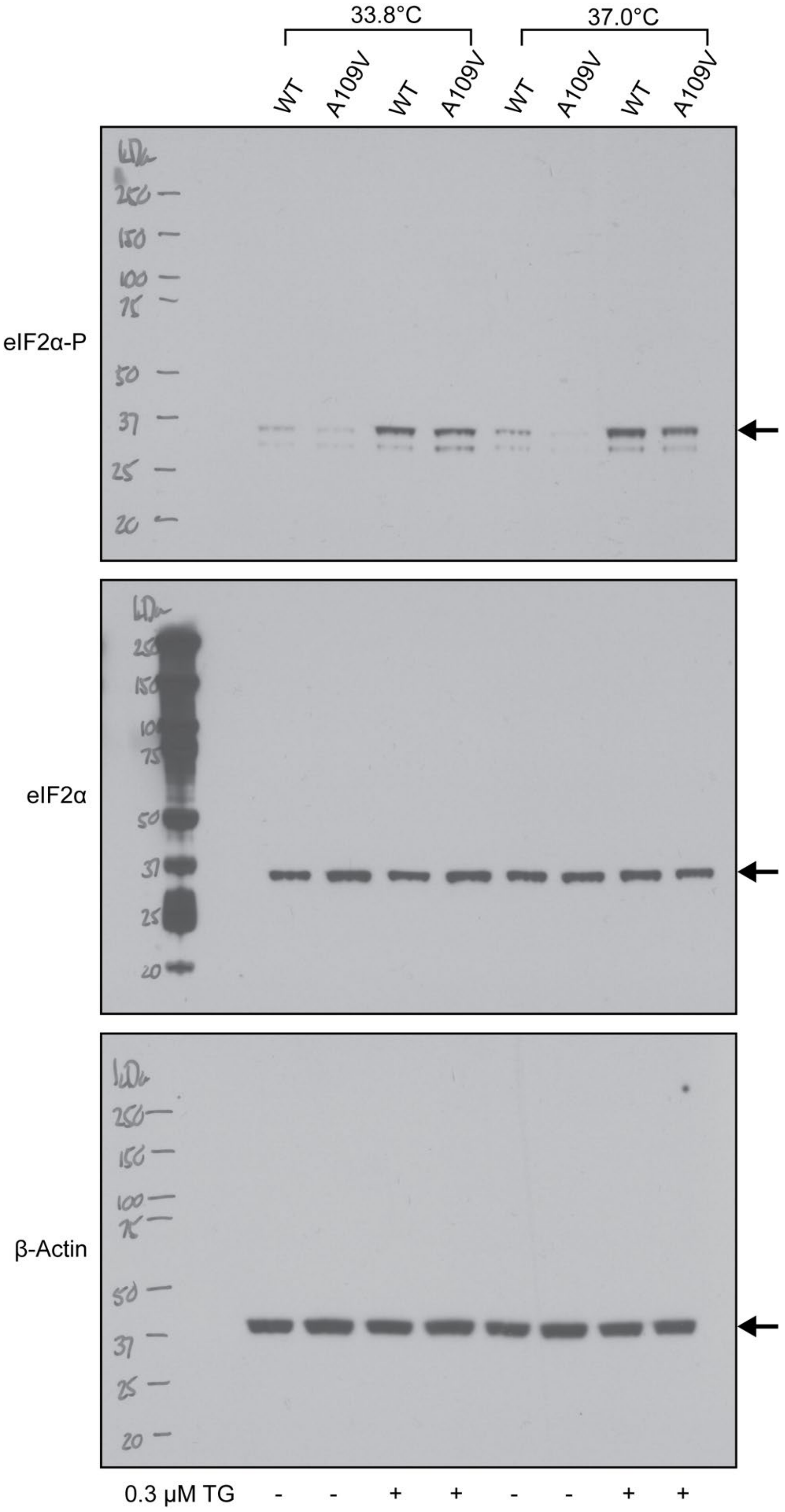

33.8°C
37.0°C
WT
A109V
WT
A109V
WT
A109V
WT
A109V
eIF2α-P
eIF2α
β-Actin
kDa
250
150
100
75
50
37
25
20
0.3 µM TG
-
-
+
+
-
-
+
+

Supplemental Figure 13. Uncropped immunoblot images to accompany Supplemental Figure 5A.

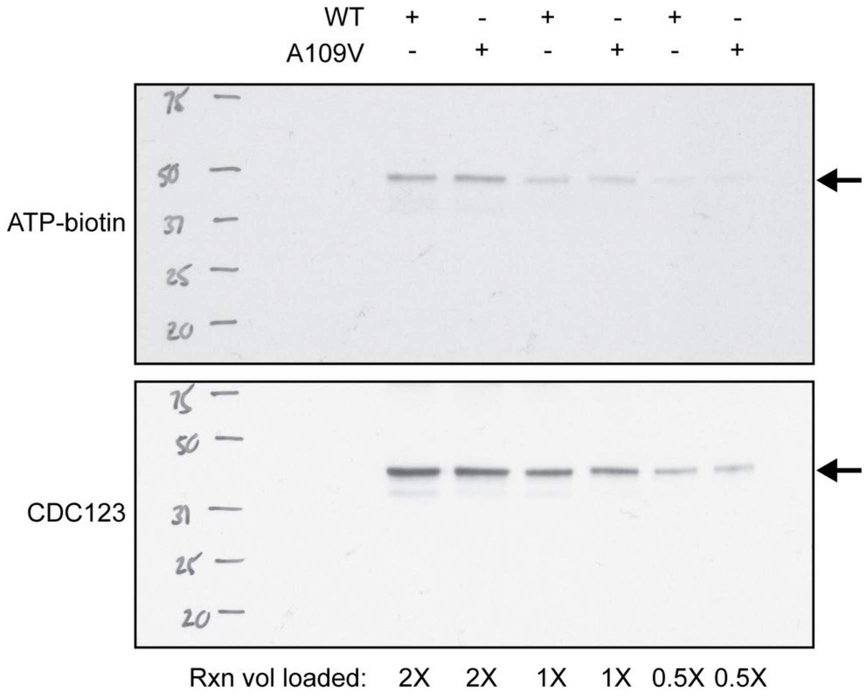

Supplemental Figure 14. Uncropped immunoblot images to accompany Supplemental Figure 5B.

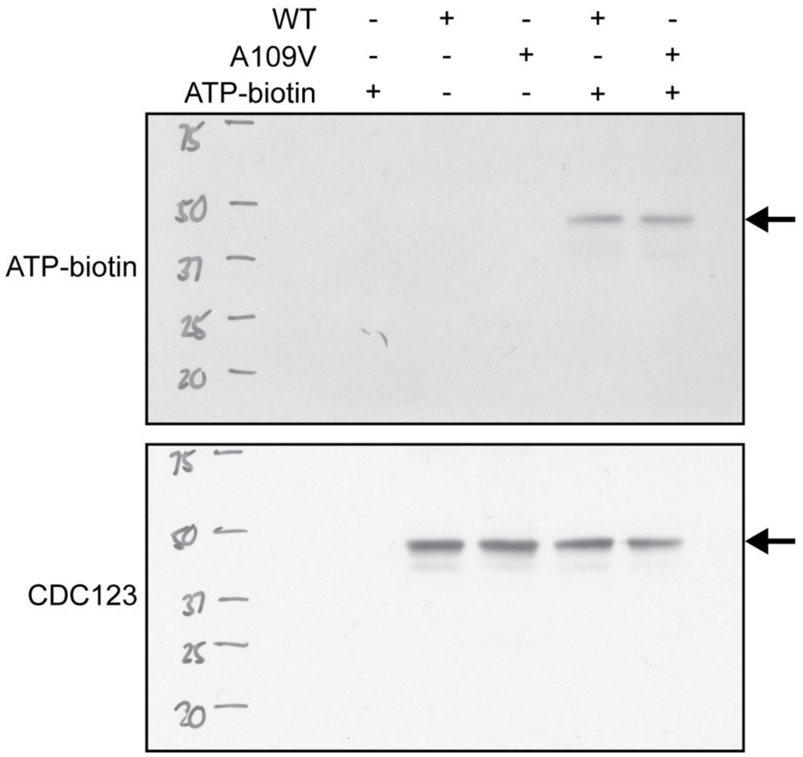

Supplemental Figure 15. Uncropped immunoblot images to accompany Supplemental Figure 5C.

Figure panel	Comparison discussed	p-value
1B	WT, 33°C versus A109V, 33°C	0.4291
1B	WT, 37°C versus A109V, 37°C	0.0005
1B	WT, 33°C versus WT, 37°C	0.0166
2C	WT, 37°C versus A109V, 37°C	0.0278
2C	A109V, 33°C versus A109V, 37°C	0.0053
2D	WT, 37°C versus A109V, 37°C	0.0036
2D	A109V, 33°C versus A109V, 37°C	0.0059
2F	WT, 37°C versus A109V, 37°C	0.0067
2F	A109V, 33°C versus A109V, 37°C	0.0045
2G	A109V, 33°C versus A109V, 37°C	0.9723
2G	WT, 33°C versus A109V, 33°C	0.0664
2G	WT, 37°C versus A109V, 33°C	0.0042
2G	WT, 33°C versus A109V, 37°C	0.0619
2G	WT, 37°C versus A109V, 37°C	0.0032
2H	WT, 37°C versus A109V, 37°C	0.0047
2H	A109V, 33°C versus A109V, 37°C	0.0044
2I	A109V, 33°C versus A109V, 37°C	0.4896
2I	WT, 33°C versus WT, 37°C	0.0037
3A	WT, NT versus WT, 0.3 μM TG	0.0024
3A	WT, NT versus WT, 1.0 μM TG	0.0002
3B	WT, 37°C, NT versus WT, 37°C, 0.3 μM TG	0.0160
3B	WT, 33°C, NT versus WT, 33°C, 0.3 μM TG	0.5052
3B	A109V, 33°C, NT versus A109V, 33°C, 0.3 μM TG	0.0076
3B	A109V, 33°C, NT versus A109V, 37°C, NT	0.0007
3B	WT, 37°C, NT versus A109V, 37°C, NT	0.0007
3B	A109V, 37°C, NT versus A109V, 37°C, 0.3 μM TG	0.7889
3C	A109V, 33°C, EV versus A109V, 37°C, EV	0.0050
3C	A109V, 37°C, EV versus A109V, 37°C, *Hs* CDC OE	0.0003
3D	A109V, 33°C, EV versus A109V, 37°C, EV	0.0024
3D	A109V, 37°C, EV versus A109V, 37°C, *Hs* CDC OE	0.0514
4C	WT versus A109V	0.0006

Supplemental Table 1. Exact p-values for data comparisons discussed in this manuscript.
